# UV–Visible–NIR camouflage textiles with natural plant based natural dyes on natural fibre against woodland combat background for defence protection

**DOI:** 10.1038/s41598-023-31725-2

**Published:** 2023-03-28

**Authors:** Md. Anowar Hossain

**Affiliations:** School of Fashion and Textiles, RMIT University, 25 Dawson Street, Brunswick, Melbourne, VIC 3056 Australia

**Keywords:** Plant sciences, Environmental sciences, Chemistry, Engineering, Materials science, Optics and photonics, Physics

## Abstract

Woodland combat background (CB) is a common source of natural plant based natural dyes (NPND). *Swietenia Macrophylla*, *Mangifera Indica*, *Terminalia Arjuna*, *Corchorus Capsularis*, *Camellia Sinensis*, *Azadirachta Indica*, *Acacia Acuminata*, *Areca Catechu and Cinnamomum Tamala* were dried-grinded-powdered-extracted-polyaziridine encapsulated-dyed-coated-printed with leafy design on cotton fabric and tested against woodland CB under the reflection engineering of ultraviolet (UV)–visible (Vis)–near infrared (NIR) spectrums and photographic versus chromatic techniques of Vis imaging. The reflection properties of NPND treated and untreated cotton fabric were experimented by UV–Vis–NIR spectrophotometer from 220 to 1400 nm. Six segments of field trialling for NPND treated woodland camouflage textiles were also investigated for concealment, detection, recognition and identification of target signature against forest plants/herbs species; common tree of woodland CB such as *Shorea Robusta Gaertn*, *Bamboo Vulgaris*, *Musa Acuminata*; and a wooden bridge made by *Eucalyptus Citriodora* & *Bamboo Vulgaris*. The imaging properties such as CIE L*, a*, b* and RGB (red, green, blue) of NPND treated cotton-garments were captured by digital camera from 400 to 700 nm against tree stem/bark, dry leaves, green leaves and dry wood of woodland CB. Therefore, a colorful matching for concealment, detection, recognition and identification of target signature against woodland CB was verified by Vis camera imaging and UV–Vis–NIR reflection mechanism. UV-protection property of *Swietenia Macrophylla* treated cotton fabric was also investigated by diffuse reflection for defence clothing. Simultaneous ‘camouflage textiles in UV–Vis–NIR’ and ‘UV-protective’ property of *Swietenia Macrophylla* treated fabric have been investigated for NPND materials-based textiles coloration (dyeing-coating-printing) which is a new concept for camouflage formulation of NPND dyed-NPND mordanted-NPND coated-NPND printed textiles in terms of ecofriendly source of woodland camouflage materials. Therefore, technical properties of NPND materials and methodologies of camouflage textile assessment have been advanced in addition to coloration philosophy of natural dyed-coated-printed textiles.

## Introduction

Natural plant based natural dyes (NPND) materials are being introduced as ‘green dyes’ and ‘green mordanting’ for natural dye-based textile coloration. NPND materials^1–6^ are common foundation of woodland combat dye for textile coloration and presently being commercialized in home and abroad, but the concept of NPND based camouflage coloration is an innovative approach of defence textiles for combat applications. The main plants/species of woodland CB are *Swietenia Macrophylla*, *Mangifera Indica*, *Terminalia Arjuna*, *Corchorus Capsularis*, *Camellia Sinensis*, *Azadirachta Indica*, *Acacia Acuminata*, *Areca Catechu*, *Cinnamomum Tamala*, *Shorea Robusta Gaertn* etc.^1^. The analytical/optical assessment technology of camouflage textiles assessments^7–11^ are still a new approach in camouflage textiles technology for defence textiles rather than field trialling method of concealment, detection, recognition and identification (CDRI) of defence target signature^12^. Presently spectral reflections of woodland CB have been considered by researchers^13,14^ but the spectral reflection versus camouflage assessment technique is still a new technology and ongoing platform of camouflage research^8,11^. Simultaneous spectrum of ultraviolet–visible–near infrared (UV–Vis–NIR) reflection is a newly applied optical technology for assessment of UV–Vis–NIR camouflage textiles technology in terms of CDRI^9^. NPND materials-polyaziridine crosslinker-UV–Vis–NIR reflection-Vis imaging versus combat engineering-color engineering-woodland CB-optical assessment-ImageJ engineering are a new approach of environment friendly materials for camouflage textiles technology, an application for defence protection^15,16^. In literature, there is enormous lacking of study related to NPND camouflage materials and its right application for camouflage textiles as nature-friendly source of NPND materials. Hence, chromatic and spectral matching were investigated between selected NPND and woodland combat background in terms of suitability for right function of camouflage materials in addition to ‘ecofriendly source’ and ‘ecofriendly processing’. Therefore, the present innovation of camouflage property has been argued for selected NPND materials against selected woodland CB. In this research of NPND-camouflage engineering, the core outcome of NPND materials against woodland CB have been hypothetically and experimentally focused without making it more comprehensive to make it more reader friendly^17^.

### Chromatic philosophy of NPND camouflage textiles against woodland CB

*Eucalyptus Radiata*, *Acacia acuminata*, *Cinnamomum tamala*, *Psidium guajava*, *Ocimum basilicum*, *Artocarpus heterophyllus*, *Terminalia Arjuna*, *Artocarpus heterophyllus*, *Terminalia bellirica*, *Areca catechu*, *Terminalia chebula*, *Swietenia macrophylla* are NPND materials applied for textile formulation and coloration. The chromatic chemistry between NPND materials and woodland CB are symmetric, hence the spectrometry, photometry & colorimetry of NPND dyed-coated-printed textiles have been assessed for CDRI against woodland CB^1,17^.

### Optical philosophy of photometry versus colorimetry for Vis camouflage textiles assessment in field trialling

RGB (red, 700 nm; green, 550 nm; and blue, 500 nm) light theory and three-dimensional color space L*, a*, b* were implemented for camouflage assessment in Vis wavelength under colorimetry of International Commission on Illumination (CIE). The grey scale and RGB scale of Vis imaging was analysed when L* = 0 signifies total absorption for perfect black, L* = 100 identifies total reflection for perfect white, + a* = red (700 nm), − a* = green (550 nm), + b* = yellow (600 nm), − b* = blue (500 nm). Therefore, saturation of color has been denoted by CIE colorimetry of red, green, blue, L*, a*, b* under day light illumination and image processing software, ImageJ^7,11,12,15,18^.

## Materials, formulations and methodology for camouflage textiles with NPND materials against woodland CB

### Exemption note of NPND materials collection and approval for textile coloration

Table 1, a minimum amount of NPND materials including ‘agricultural waste’^19^ were collected from non-commercial/commercial sources applied for non-commercial application. NPND application for camouflage textiles is an academic platform of research to propose a practicability for broader scale implementation in terms of ecofriendly source of camouflage materials. All experiment of NPND materials were conducted with accordance to relevant regulations and guidelines. Therefore, permission of NPND sample collection is not required. This exemption is supported by ‘3.1.2, assessment requirement’ for collection of NPND materials quoted as below, registered research located in Australia.

**Table 1:** Experimental applications of selected NPND-materials for three-dimensional process of camouflage formulation and coloration (dyeing-coating-printing) and selected forest plants/herbs for comparison with woodland CB.

Scientific name	Local name of sample collection zone/field trialling, Bangladesh	Used parts of tree in this experiment	Purpose of NPND in this experiment
*Swietenia macrophylla*	Mahogany tree	Wood	Dyeing-coating-printing
*Mangifera indica*	Mango tree	Leaves	Dyeing
*Terminalia arjuna*	Arjun tree	Barks	Dyeing
*Corchorus capsularis*	Jute plant	Leaves	Dyeing-coating-printing
*Camellia sinensis*	Tea plant	Modified leaves powder	Dyeing
*Azadirachta indica*	Neem tree	Leaves	Dyeing
*Acacia acuminata*	Jam/Peach tree	Seeds	Dyeing
*Areca catechu*	Areca Palm/Supari tree	Seeds	Dyeing, Bio-Mordanting
*Cinnamomum tamala*	Pecker tree	Leaves	Dyeing
*Shorea Robusta gaertin*	Sal/Gajari	A group of trees in a forest area included tree stem/bark, green leaves, dry leaves	Woodland CB
*Bamboo vulgaris*	Bamboo tree	Woody stem for wooden bridge	Woodland CB
*Eucalyptus citriodora*	Eucalyptus tree	Wood for wooden bridge	Woodland CB
*Bamboo vulgaris*	Bamboo tree	A group of trees included tree stem/bark, mostly green leaves	Woodland CB
*Musa acuminata*	Banana tree	A group of trees included tree stem/bark, mostly green leaves	Woodland CB

“A permit is not required to collect native plant material from private land, unless: the species for collection are listed threatened species or form part of a threatened ecological community under the environment protection and biodiversity conservation (EPBC) Act, under such circumstances a Commonwealth permit may be required”^20^.

### Experimental preparations in general

Table 1, nine-NPND materials of woodland CB were collected, washed, dried, powdered/grinded and extracted by water medium. Three-dimensional method of textile coloration was applied to signify the feasibility of NPND-camouflage coloration of dyeing, coating and printing^21–23^. In terms of maximum and minimum reflection, standardized white and black background was used for comparison between target object, dyed-coated-printed textiles and selected woodland CB.

### Coating and printing with NPND on cotton fabric

**Figure 1:**
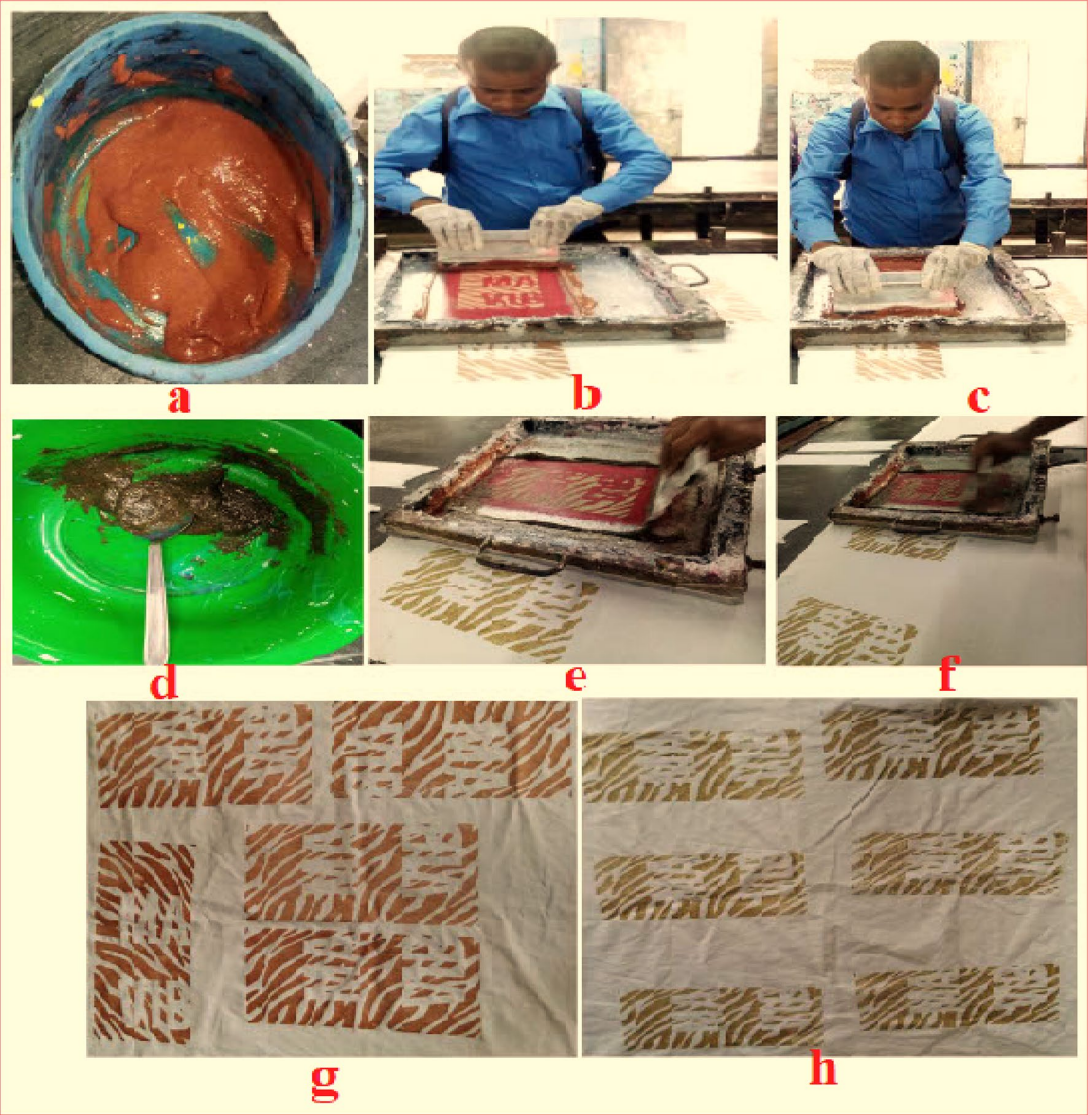
Preparation and laboratory process for coating and printing with NPND-*Swietenia Macrophylla* and NPND-*Corchorus Capsularis*.

Table 2, a composite paste of Tubassist Fix 104W, a crosslinking agent of polyaziridine^24^; NK binder, acrylonitrile copolymer^25,26^; F53 glitter paste, acrylic copolymer and selected NPND-powder was formulated for coating and printing paste. Therefore, S/J knitted cotton fabric was coated with wooden scrubber and printed with rubber scrubber. Figure 1 identifies the complete coloration/preparation process of printing. Figure, 1a shows the formulated recipe of polyaziridine encapsulated NPND-*Swietenia Macrophylla*^22,24,27,28^; Fig. 1b and c means the trialling conditions of printing; Figure, 1d shows the formulated recipe of grinded dry leaves of polyaziridine encapsulated NPND^24^-*Corchorus Capsularis*; Fig. 1e and f means the trialling conditions of NPND-*Corchorus Capsularis* printing on cotton fabric; Fig. 1g and h shows the pictorial image of NPND-*Swietenia Macrophylla* and NPND-*Corchorus Capsularis* printed fabric after curing.

**Table 2:** Chemicals and formulations for coating and printing with NPND materials on cotton fabric.

Name of chemicals	Company	A common formulated recipe was trialed for coating and printing	Fixation
Tubssist Fix 104W	CHT	10–15%	Sun light fixation 30 min; temperature (30–40) °C
NK binder	E-chem	5–10%	
F53 glitter paste	E-chem	5–10%	Heat fixation 2–4 min; temperature (100–120) °C
NPND-powder	–	75–65%

### Mordant free NPND-coloration and NPND-mordanting for natural dyeing with NPND materials on natural fibre

Nine individual NPND was formulated for mordant free coloration on cotton fabric for camouflage textiles. An exhaust method of NPND coloration process was applied for camouflage coloration on cotton fabric. 150 GSM, 100% cotton knitted fabric (scoured, bleached) was washed with 2% detergent. 5 g powdered *Swietenia Macrophylla* (wood/stem) was boiled with 1.5 L water at boiling temperature, run time 120 min in an open bath and continuous method. 10 g cotton fabric was dyed for 90 min in an open bath at boiling temperature and followed a continuous method of coloration. A post mordanting technique was applied with the seed of *Areca catechu* in terms of NPND-mordanting^19^. Similarly, a mordant free coloration with processed tea leaves was continued by water medium: water 2.5 L, processed black tea 25 g, Taza, Uniliver Bangladesh Ltd.; extraction, and dyeing time 120 min in an open bath at boiling temperature and followed a continuous method of coloration. Correspondingly, *Mangifera indica* (leaves), *Terminalia arjuna* (bark), *Corchorus Capsularis* (leaves), *Camellia sinensis* (modified leaves powder), *Azadirachta indica* (leaves) with *Areca Catechu* (NPND-mordant/bio-mordant), *Acacia acuminata* (seed), *Cinnamomum Tamala* (leaves) were formulated for NPND dyeing on cotton fabric, separately noted in Tables 1 and 2.

### Field trialling segment: 1–6 for camouflage textile assessment against woodland combat background under natural illumination of day light

Practically, visual acuity is the camouflage physics of human perception for visual assessment of CDRI for defence target signature which can be denoted by α = Y/L where α = visual acuity, Y = size of target signature/object, NPND dyed-coated-printed garment and L = distance between object (dyed-coated-printed textiles) and observer/surveillance device^15,29^. Hence, field trialling of NPND dyed-coated-printed garments was conducted against black and white standard. NPND dyed-coated-printed garments were placed against woodland, wooden bridge, soil bed, concrete bed. Nikon Coolpix s2500, Nikon Corporation, Japan; 74,163,447 was used to capture Vis-photograph under selected distance. A constant measurement of coated and printed garments size was applied for whole experimentation of digital image^29,30^. Photographs of NPND dyed, coated and printed cotton fabric in the form of stitched garments against woodland CB were captured and considered as real image of object-background reflectance in visible range. Chromatic deviations of target signature against woodland CB were analysed by three dimensional color space mechanism of CIE L* = 0 (black), L* = 100 (white), + a* (red), − a* (green), + b* (yellow), − b* (blue) and RGB (red, green, blue) trichromatic technique of grey (white–black) chroma saturation^18^. Captured RGB images were converted into RGB stack and CIE L*, a*, b* stack for camouflage assessment of individual chromatic hue of CIE red, green, blue, L*, a*, b* against multidimensional CBs. Image functions of ImageJ software were applied for this assessment process. CIE L*, a*, b* values are generated by ImageJ for chromatic values from o to 100 where 0 represents black and 100 represents white. RGB values are also generated by ImageJ software for pixel range from 0 to 255 where 0 represents black and 255 represents white^7^. L*, a*, b*, RGB values of NPND textiles against CBs are generated under ‘light trapping’ limitation of natural illumination, sunlight^12^. The imaging of ‘light trapping’ is considered as an uncontrollable condition of natural illumination for field trialling.

### Field trialling segment: 1–3 for camouflage textiles against woodland CB, *Shorea Robusta Gaertn*

This experimentation was trialled against woodland CB, *Shorea Robusta Gaertn* during February–March 2022 under natural illumination (sunlight) of morning day light in a forest environment for CDRI of defence target signature^12^. A garment of NPND-nine color combination of NPND materials cited in Table 1; a single color coated and printed garment with *Swietenia Macrophylla*; a single color coated and printed garment with *Corchorus Capsularis* were trialled for field experimentation of camouflage target signature against woodland CB. The distance between target textile sample and camera was kept 7 ft and 21 ft for field trialling. For chromatic assessment, 736 × 850 pixels, RGB and CIE L*, a*, b* image were generated by ImageJ software.

### Field trialling segment: 4–6 for camouflage textiles against woodland CB; *Musa Acuminata*,* Bamboo Vulgaris,* wooden bridge made by *Eucalyptus Citriodora* and *Bamboo Vulgaris*; and concrete CB

Dry wood of *Eucalyptus Citriodora*, Bangladesh origin was purchased from three different tree and three different ages of tree including 40–50% heartwood. For this experimentation, three types of common forest tree were selected as woodland CB such as *Eucalyptus Citriodora*, *Bamboo Vulgaris* and *Musa Acuminata*. A wooden bridge was constructed by the combination of dry wood of *Eucalyptus Citriodora* and *Bamboo Vulgaris*^15^. This image of dyed-coated-printed textiles captured against a constructed wooden bridge made by *Eucalyptus Citriodora*, *Bamboo Vulgaris*; and woodland CB of *Bamboo Vulgaris* and *Musa Acuminata*. This woodland background additionally covers soil and concrete CB. Images were captured by digital camera under natural illumination of day light on 19 January, 2023 at 2–3 pm. A garment of NPND-nine color combination of NPND materials cited in Table 1; a single color coated and printed garments with *Swietenia Macrophylla*; a single color coated and printed garments with *Corchorus Capsularis* were trialled for field experimentation of camouflage target signature against woodland CB. The distances between target textile sample and camera were kept 6 ft and 22 ft for field trialling. For chromatic assessment, 3161 × 3807 pixels, RGB and CIE L*, a*, b* images were generated by ImageJ software.

### Fabrication and groundwork process of field trialling against woodland CB; NPND treated fabrication and field trialling of nine color combination

**Figure 2:**
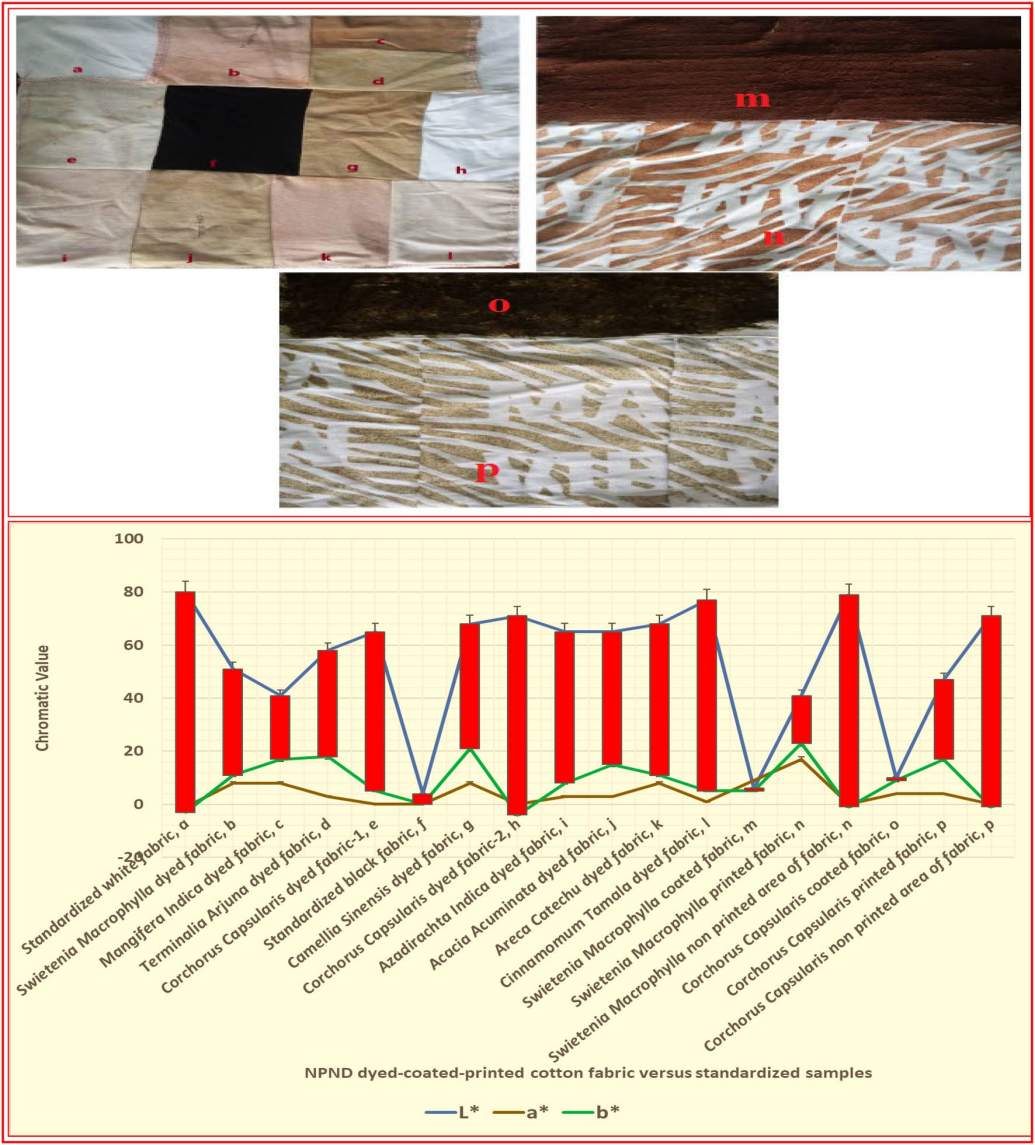
NPND dyed cotton fabric with solid color design compared with standardized white and black fabric (**a**–**l**), NPND coated cotton fabric with solid color design (**m**, **o**) and NPND printed cotton fabric with leafy design (**n**, **p**) including chromatic value of CIE L*, a*, b*.

Figure 2a–l, fabrication of nine color combination^31^ of NPND against standardized black and white fabric as low reflection and high reflection chromatic hue. a = standardized white fabric, b = *Swietenia Macrophylla* dyed fabric, c = *Mangifera Indica* dyed fabric, d = *Terminalia Arjuna* dyed fabric, e = *Corchorus Capsularis* dyed fabric-1, f = standardized black fabric, g = *Camellia Sinensis* dyed fabric, h = *Corchorus Capsularis* dyed fabric-2, i = *Azadirachta Indica* treated fabric with *Areca Catechu* as NPND-mordanting/bio-mordanting, j = *Acacia Acuminata* dyed fabric, k = *Swietenia Macrophylla* treated fabric with *Areca Catechu* as NPND-mordanting/bio-mordanting, l = *Cinnamomum Tamala* dyed fabric. The chromatic values (L*, a*, b*) of NPND dyed fabric and standardized fabric (a–l) have been captured by ImageJ software and shown accordingly for chromatic comparison. Hence, nine color-NPND was dyed on cotton fabric, cut in small pieces and stitched by cotton yarn for measurement of optical reflection under natural illumination of day light and Vis imaging principle against woodland CB^32^.

### NPND-*Swietenia Macrophylla* coated and printed fabrication with single color chromatic hue

Figure 2m–n, *Swietenia Macrophylla* wood powder was coated with solid color design and printed with single color leafy design matching with woodland CB. The chromatic values (L*, a*, b*) of *Swietenia Macrophylla* coated and printed fabric have been captured by ImageJ software and shown accordingly for chromatic comparison.

### NPND-*Corchorus Capsularis* coated and printed fabrication with single color chromatic hue

Figure 2o and p, *Corchorus Capsularis* leaves powder was coated^33^ with solid color design and printed with single color leafy design for woodland CB. The chromatic values (L*, a*, b*) of *Corchorus Capsularis* coated and printed fabric have been captured by ImageJ software and shown accordingly for chromatic comparison.

## Testing and measurement process for camouflage textiles assessment with UV–Vis–NIR spectrophotometer

### Kubelka–Munk (K–M) diffuse theory of UV–Vis–NIR reflectance spectroscopy

Equation (1), validation of diffuse reflectance using Kubelka–Munk (K–M) theory of scattering between target signature and woodland CB. For camouflage assessment in laboratory stages, K–M theory is a theoretical reference for diffuse reflectance in UV–Vis–NIR spectrum.

1 $$\mathrm{K}/\mathrm{S }= \frac{{(1-\mathrm{R\infty })}^{2}}{2\mathrm{R\infty }}$$

Reflectance (R) is inversely proportional to the light scattering co-efficient (S) and s value is inversely proportional to the particle size of treated textiles and the materials of woodland CB^7^.

### Sampling procedure for assessment with UV–Vis–NIR spectrophotometer

**Figure 3:**
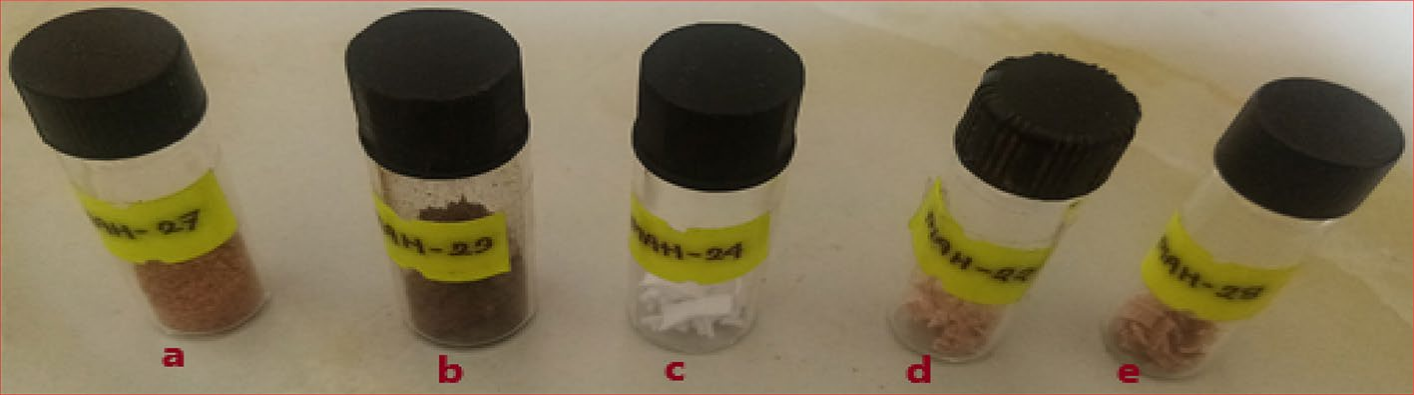
Samples of NPND-powdered materials (**a**, **b**); cut pieces of undyed cotton fabric (**c**) and cut pieces NPND-dyed cotton fabric (**d**, **e**) for measurement with sample port of UV–Vis–NIR spectrophotometer.

Sampling procedure for measurement in UV–Vis–NIR spectrum was conducted under constant reflection of barium sulfate as 100% reflectance materials. Powdered sample and round cut of fabric was placed on standardized glass vial for reflection spectra. In Fig. 3a = powder of *Swietenia Macrophylla*, Fig. 3b = powder of *Areca Catechu*, Fig. 3c = cut pieces of raw knitted fabric before dyeing, Fig. 3d = cut pieces of *Swietenia Macrophylla* dyed fabric without mordanting, Fig. 3e = cut species of *Swietenia Macrophylla* dyed fabric with *Areca Catechu*-mordanting.

### Scanning condition of UV–Vis–NIR spectrophotometer

**Figure 4:**
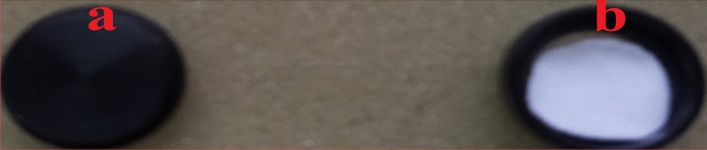
Blank sample port (**a**) and standardized barium sulphate placed on sample port (**b**) for assessment with UV–Vis–NIR spectrophotometer.

UV–Vis–NIR spectrophotometer, named UV-2600 spectrophotometer was used for diffuse reflectance from 200 to 1400 nm. This machine was a set-up with integrating sphere. A film plate of standardized barium sulphate was scanned as reference of 100% reflection material in room temperature shown in Fig. 4.

## Results and discussion

### Assessment of UV–Vis–NIR camouflage properties of *Swietenia Macrophylla* dyed fabric against woodland CB materials for concealment of target signature

**Figure 5:**
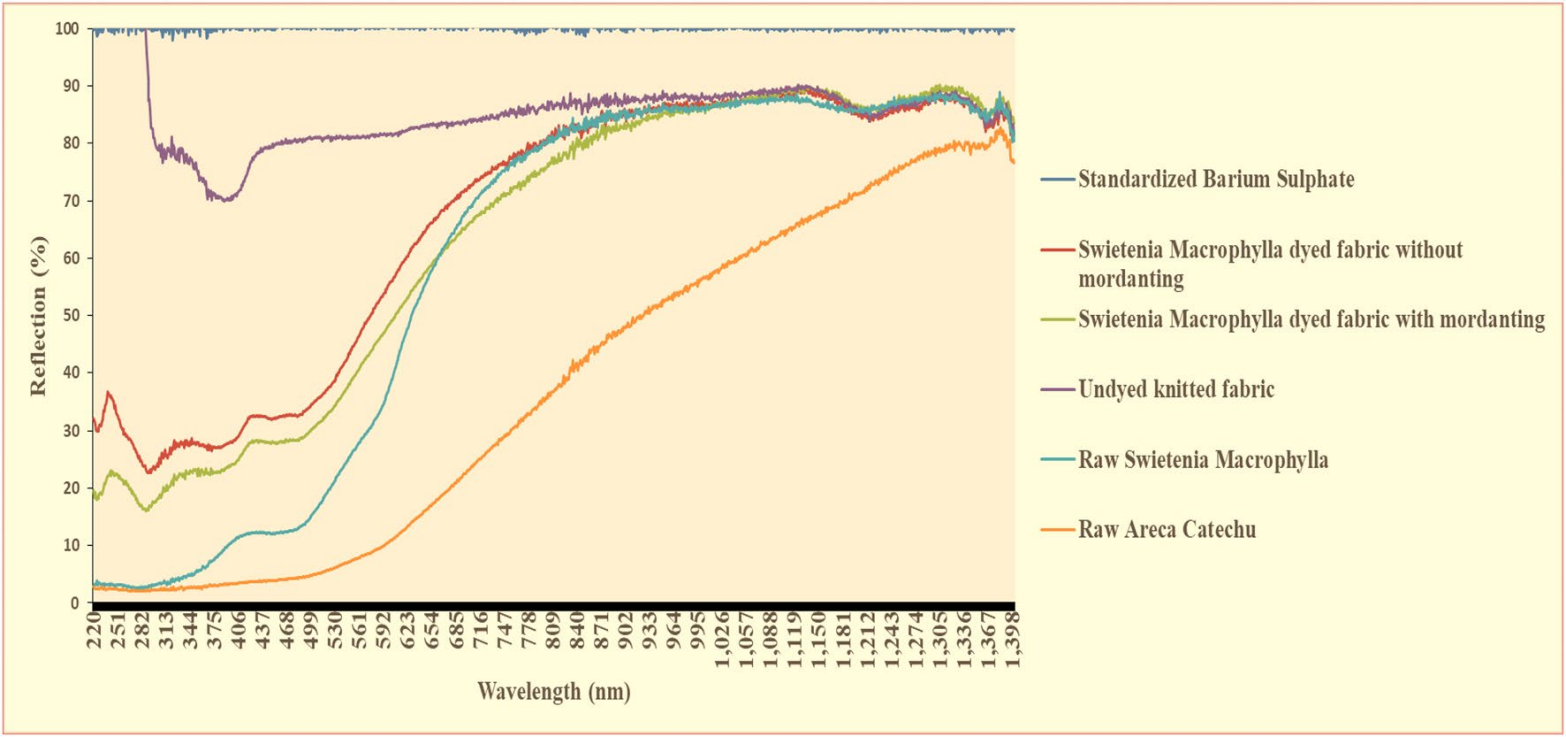
UV–Vis–NIR reflection spectra of undyed cotton fabric, dyed cotton fabric and NPND raw materials of woodland CB against standardized barium sulphate.

Figure 5, the reflection% of UV–Vis–NIR spectrum obtained from the standardized barium sulphate, *Swietenia Macrophylla* dyed fabric without mordanting, *Swietenia Macrophylla* dyed fabric with NPND-mordanting and undyed knitted fabric against the materials of woodland CB. The reflection% of raw *Swietenia Macrophylla* and raw *Areca Catechu* was scanned as the materials of NPND and woodland CB. The reflection deviation of UV spectrums from 220 to 375 nm are found from 5 to 35% but the reflections were found symmetrical. The variation of reflections in Vis from 412 to 685 nm are remarked as 5% to 60% but the reflections were shown symmetrical. The NIR reflections are recorded from 25 to 80% from 716 to 1398 nm. A symmetrical C–O and O–H group stretching have been exhibited from 1305 to 1398 nm and 778 nm to 871 nm. The C–O strength is comparatively more vibrated in NIR spectrums. For chromatic explanation of UV to Vis range, the deviation of chromatic reflections are minor in UV and Vis region due to optical scattering and chromatic matching in terms of established chromatic assessment in Vis region related to violet (400–450 nm), indigo (450–500 nm), green (500–550 nm), yellow (550–600 nm), orange (600–650 nm) and red (650–700 nm). Chromatic intensity by reflection% in UV to Vis range from 220 nm 592 nm are comparatively lower due to higher absorption. The reflection% are gradually increased from 530 to 871 nm. From 901 nm, chromatic reflection starts to stabilize into straight direction due to lower absorption under continuous stretching vibrations. The key features of reflection% have been found a harmonized direction in the entire area of graphical stage from 220 to 1398 nm. Hence the entire reflection% of *Swietenia Macrophylla* dyed fabric have been demonstrated a graphical matching in UV–Vis–NIR compared with raw *Swietenia Macrophylla* and raw *Areca Catechu* which are signified as camouflage property under symmetrical properties of woodland CB. The compositional structures of cellulosic cotton and cellulosic structure of woodland CB have also been found symmetry in NIR reflection% from 716 to 1398 nm. The graphical line of reflection% of raw *Areca Catechu* was comparatively straight due to hard surface of *Areca Catechu* particles. Hence the darker chromatic hue of reddish tone of *Swietenia Macrophylla* wood (inner side) coated/dyed/printed fabric is correlated with the *Swietenia Macrophylla* tree of woodland CB due to similarities of existing phytochemical properties of chromatic reflection such as tannin, lignin, flavonoids etc. The molecular vibration of *Swietenia Macrophylla* in NIR has been excited associated with C=O, C–H, O–H and N–H bonds due to presence of lignin, cellulose, hemicellulose and phenolic compound. Hence the stretching vibrations are comparatively increased in NIR rather than UV–Vis range. The symmetrical stretching of O–H bond has been found due to cellulosic properties of *Swietenia Macrophylla*-wood and cotton fabric. Consequently, C=O and C–H stretching were also observed due to existing hemicellulose both in cotton fibre and *Swietenia Macrophylla*^34^. The supporting information of UV–Vis–NIR camouflage assessment from 220 to 1400 nm have been sequentially attached in Tables 1–12 which are remarked as reflection (%) of standardized barium sulphate, *Swietenia Macrophylla* dyed fabric without mordanting, *Swietenia Macrophylla* dyed fabric with mordanting, undyed knitted cotton fabric, raw *Swietenia Macrophylla* and raw *Areca Catechu*.

### Optical assessment of UV-protection property of *Swietenia Macrophylla* dyed fabric

**Figure 6:**
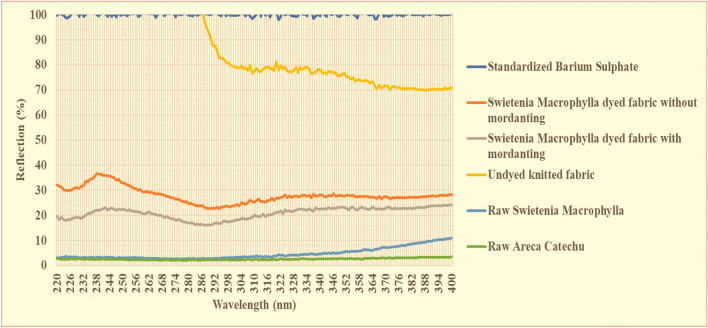
UV-reflection of *Swietenia Macrophylla* dyed fabric with NPND mordanting and without mordanting, untreated cotton fabric, raw *Swietenia Macrophylla* and raw *Areca Catechu* compared with standardized barium sulphate.

Figure 6; possibly, the reflection of raw cotton fibre is critically uncommon due to high exposure of UV light from 220 to 282 nm. It may happen because of acceleration of yellowness and whiteness chroma, increased light energy, therefore white fabric looks exceptionally brighter, hence the reflection tends to accelerate more than 100%. The UV parameters from 220 to 406 nm were also remarked based on UV reflection spectra^35^. UV protection of UV-A and UV-B are comparatively lower of *Swietenia Macrophylla* treated fabric due to minimum percentage of reflectance, from 20 to 30%. Optical properties of light are related to transmission, absorption and reflection. According to established theory of energy, absorptivity + reflectivity + transmittance = 1. Therefore, the total expected absorption and transmittance will be around 70%. Natural illumination of UV is generally classified by UV-A (315–400 nm), UV-B (280–315 nm) and UV-C (below 280 nm) although UV-C was not measured in this experiment. The reflection% of UV-A and UV-B of *Swietenia Macrophylla* treated fabric were analysed by UV spectrum exposed in Fig. 6. The significant variations of UV reflection were observed between NPND dyed fabric and undyed knitted cotton fabric. UV protection of *Swietenia Macrophylla* dyed fabric was found 20% to 30%. The supporting information of UV-optical properties have been attached in Tables 1 and 2.

### Field trialling segments: 1–3; chromatic analysis of NPND dyed-coated-printed garments, target signature against woodland CB, *Shorea Robusta Gaertn* under natural illumination of day light

**Figure 7:**
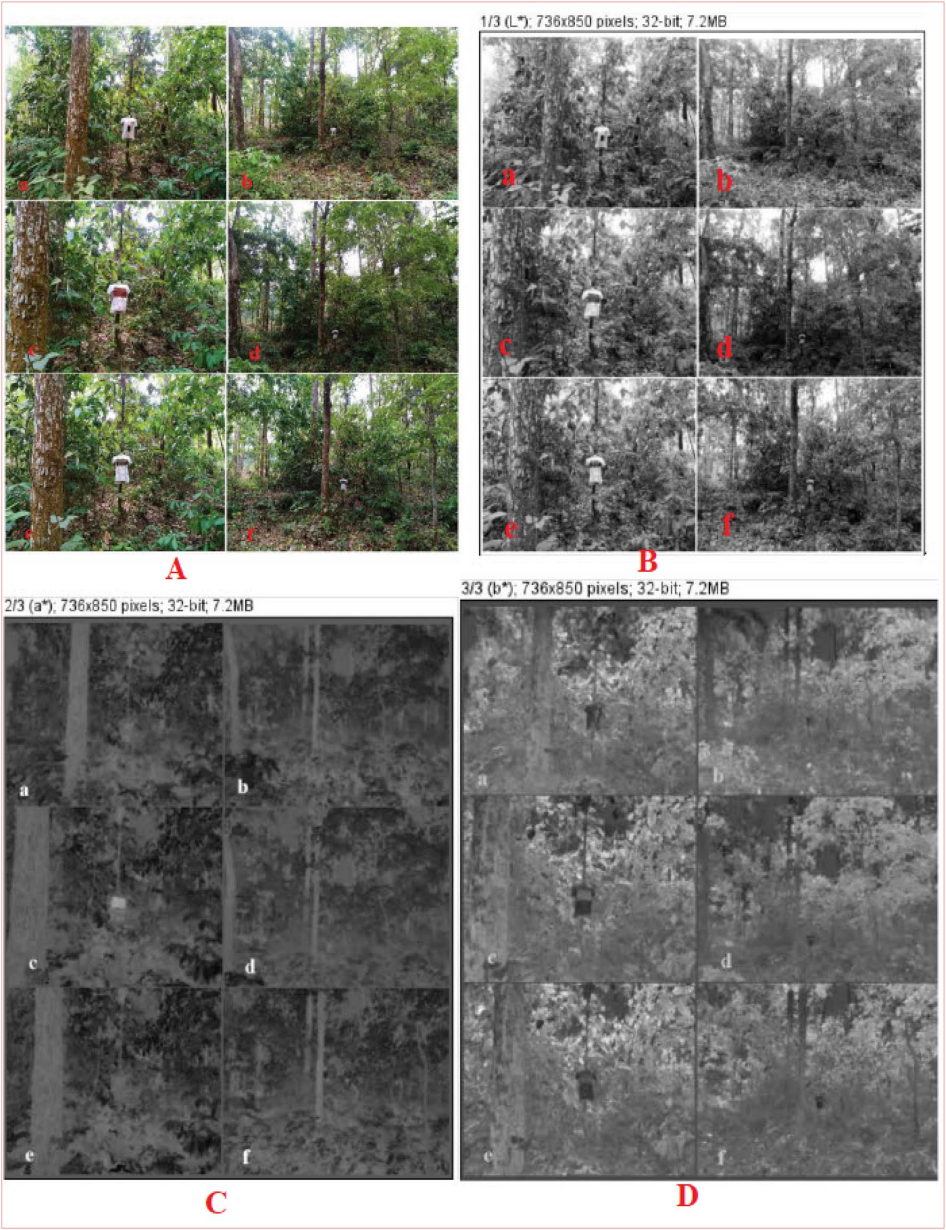
NPND-dyed-coated-printed garments placed against woodland CB; shown the photometric versus colorimetric views of CIE L* (**B**), a* (**C**), b* (**D**) in grey scale imaging of RGB raw image (**A**) when the pictorial mapping has been engendered as Aa = Ba = Ca = Da; Ab = Bb = Cb = Db; Ac = Bc = Cc = Dc; Ad = Bd = Cd = Dd; Ae = Be = Ce = De; Af = Bf = Cf = Df for chromatic comparison.

**Figure 8:**
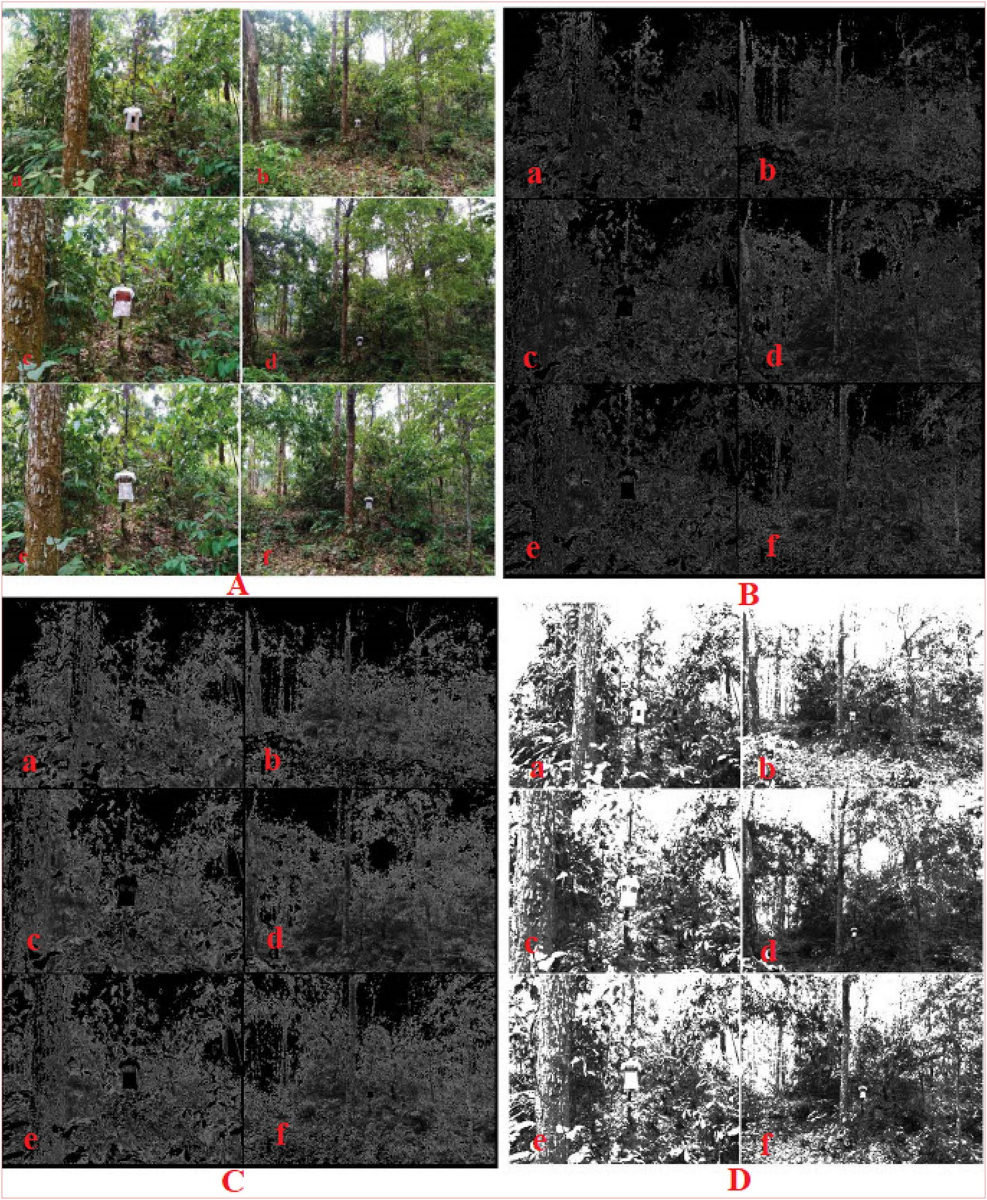
NPND-dyed-coated-printed garments placed against woodland CB and shown the photometric versus colorimetric views of blue (**B**), green (**C**), red (**D**) in grey scale imaging of RGB raw image (**A**) when the pictorial mapping has been engendered as Aa = Ba = Ca = Da; Ab = Bb = Cb = Db; Ac = Bc = Cc = Dc; Ad = Bd = Cd = Dd; Ae = Be = Ce = De; Af = Bf = Cf = Df for chromatic comparison.

**Figure 9:**
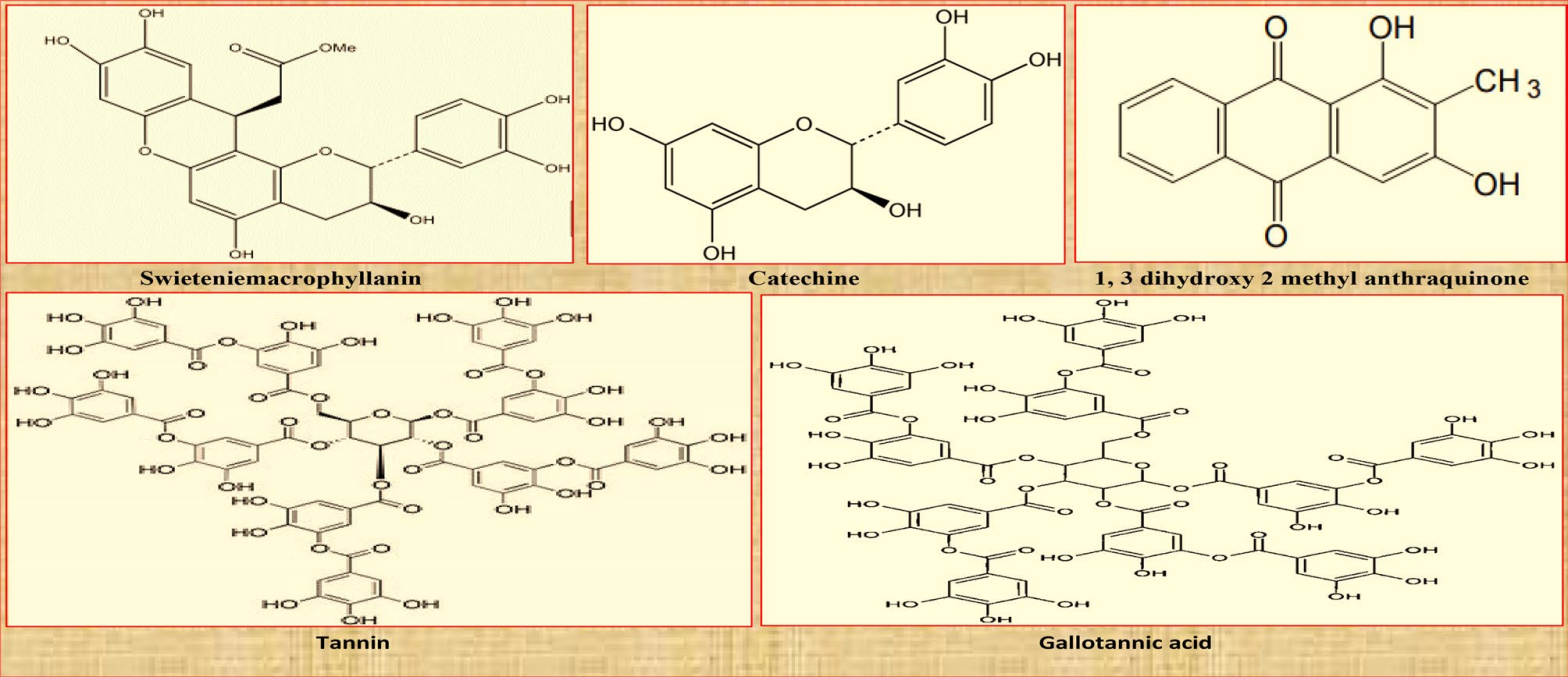
Phytochemicals of NPND materials responsible for camouflage coloration against woodland CB, *Shorea Robusta Gaertn.*

Figure 7A, raw image of NPND-dyed-coated-printed garments have been placed against *Shorea Robusta Gaertn*, woodland CB. Figure 7Aa and Ab depicts target signature of dyed fabric with nine color combination of NPND. Figure 7Ac and Ad remarks target signature of *Swietenia Macrophylla* coated and printed fabric. Figure 7Ae and Af shows the target signature of *Corchorus Capsularis* coated and printed fabric. Therefore, a combat engineering-color engineering-digital camera imaging-image processing technique was applied for Vis-range camouflage textiles assessment against selected CB environment. The target signatures were captured from two selected distances and the pictorial mapping were signified in Figure as 7Aa = 7Ac = 7Ae = 7 ft and 7Ab = 7Ad = 7Af = 21 ft for chromatic variation versus CDRI. Under CDRI evaluation; 7Ab, 7Ad, 7Af located target signature have been found partially camouflage against the materials of woodland CB such as tree stem/bark, green leaves, dry leaves of *Shorea Robusta Gaertn*. Accordingly; 7Aa, 7Ab, 7Ac, 7Ad, 7Ae, 7Af located target signature have been found color matching against woodland CB. Figure 7Aa and Ab, the reflection mechanism of dyed fabric with nine color combination was almost matched with woodland CB (*Shorea Robusta Gaertn*) compared with standardized black and white color fabric. The chromatic hue of NPND-nine color combination has minor difference of reflection compared with tree stem/bark, green leaves, dry leaves of *Shorea Robusta Gaertn*. Therefore, NPND-dyed garment has been found minor difference of chromatic reflection against woodland CB under natural illumination of day light. Figure 7Ac and Ad; the reddish chromatic hue of *Swietenia Macrophylla* coated part of garment was completely matched with the combat bark of *Shorea Robusta Gaertn* due to matching of phenolic pigment, anthocyanin of *Swietenia Macrophylla* treated textiles. Figure 7Ae and Af, the coated and printed fabric with *Corchorus Capsularis* was trialled with field experimentation against *Shorea Robusta Gaertn*, woodland CB and the target signature was also remarked as chromatic matching against woodland CB. The greenish chroma of *Corchorus Capsularis* coated part of garment was completely matched with the green leaves of *Shorea Robusta Gaertn*, possibly the well matching of green pigment named chlorophyll compound. Furthermore, existing protochlorophyll, anthocyanin and catechin in NPND may be the vital reason of reflection matching between woodland CB and target object of coated/printed NPND textiles. Figure 7B–D are remarked for the chromatic hue of CIE L*, a*, b*; generated from raw image mentioned in Fig. 7A. The mapping of Figure has been signified as Aa = Ba = Ca = Da; Ab = Bb = Cb = Db; Ac = Bc = Cc = Dc; Ad = Bd = Cd = Dd; Ae = Be = Ce = De; Af = Bf = Cf = Df; shown in Fig. 7 for photometric and colorimetric comparison of L*, a*, b*. Therefore, white-black/grey scale breakdown of CIE L*, a*, b* of object and background have been found camouflage shown in Fig. 7Ba,Ca,Da,Bb,Cb,Db,Bc,Cc,Dc,Bd,Cd,Dd,Be,Ce,De,Bf,Cf,Df and compared with raw image of target signature against *Shorea Robusta Gaertn*, woodland CB shown in Fig. 7Aa,Ab,Ac,Ad,Ae,Af. Similarly Fig. 8A; raw image of NPND-dyed-coated-printed garments have been positioned against *Shorea Robusta Gaertn*, woodland CB; and RGB chromatic hues have been represented as blue color shown in Fig. 8B, green color shown in Fig. 8C and red color shown in Fig. 8B when Aa = Ba = Ca = Da; Ab = Bb = Cb = Db; Ac = Bc = Cc = Dc; Ad = Bd = Cd = Dd; Ae = Be = Ce = De; Af = Bf = Cf = Df shown in Fig. 8. White-black/grey scale breakdown of RGB chromatic hue has also been shown camouflage shown in Fig. 8Ba,Ca,Da,Bb,Cb,Db,Bc,Cc,Dc,Bd,Cd,Dd,Be,Ce,De,Bf,Cf,Df compared with raw image of target signature against woodland CB revealed in Fig. 8Aa,Ab,Ac,Ad,Ae,Af. Figures 7Aa,Ab,Ba,Bb,Ca,Cb,Da,Db and 8Aa,Ab,Ba,Bb,Ca,Cb,Da,Db; dry leaves, seeds, tree bark and wood of NPND materials have presence of tannin (polyphenolic compound) which creates strong complex with OH group of cellulosic cotton fabric. Tannase is the main element of tannin. Tannase may be responsible for matching NPND-color formation of camouflage textile applications against woodland CB^36^. The chromatic reflection of dry leaves, green leaves, tree bark and wood of woodland CB, *Shorea Robusta Gaertn* are related to chlorophyll/phytochemical in terms of green pigmentation/reddish chromatic hue^37^. The reflection of NPND dyed textiles have been found almost similar reflection of woodland CB as dry leaves, green leaves, tree bark and wood are the key materials of woodland CB, *Shorea Robusta Gaertn*^36,37^. Therefore, possible chromatic matching of phytochemicals exists between NPND dyed-coated-printed garment and woodland CB, *Shorea Robusta Gaertn*. These phytochemicals are classified as swieteniemacrophyllanin; catechine; 1,3 dihydroxy 2 methylanthraquinone; tannin and gallotannic acid which are chemically structured in Fig. 9. Furthermore, *Areca Catechu* is a NPND-mordanting agent which has the property of color sensitizing. *Areaca Catechu* has coloring agents and color improvement agents due to remaining tannin, gallic acid, catechin, alkanoids, gum, etc. Existing gallotannic acid in *Areca Catechu* is a pigment, it may be responsible for color sensitizer as NPND-mordanting for NPND applications on textile substances^38,39^. Figures 7Ac,Ad,Bc,Bd,Cc,Cd,Dc,Dd and 8Ac,Ad,Bc,Bd,Cc,Cd,Dc,Dd; the reddish chromatic hue of *Swietenia Macrophylla* is related to “reddish pale compound” named swieteniemacrophyllanin has the tendency for reddish chromatic hue of coated cotton textiles as source of NPND. *Swietenia Macrophylla* coated fabric has been found exact matching against woodland CB, *Shorea Robusta Gaertn*^40^. Similarly, Figs. 7Ae,Af,Be,Bf,Ce,Cf,De,Df and 8Ae,Af,Be,Bf,Ce,Cf,De,Df; “greenish hue” of *Corchorus Capsularis*-dry leaves has also been observed color matching against woodland CB, *Shorea Robusta Gaertn* due to matching of chlorophyl and existing phytochemicals such as tannin and catechine of polyphenol group. Hence a crosslinking of NPND powder-polyaziridine-acrylonitrile copolymer-acrylic copolymer-cellulose may be formed to create a symmetrical chromatic hue of coated and printed textiles against woodland CB. This NPND formulation can be implemented for nature-friendly source of camouflage materials.

### Field trialling segments: 4–6; chromatic analysis of NPND dyed-coated-printed garments, target signature against woodland CB, *Bamboo Vulgaris, Musa Acuminata*; and wooden bridge made of* Eucalyptus Citriodora*,* Bamboo Vulgaris*; and concrete CB under natural illumination of day light

**Figure 10:**
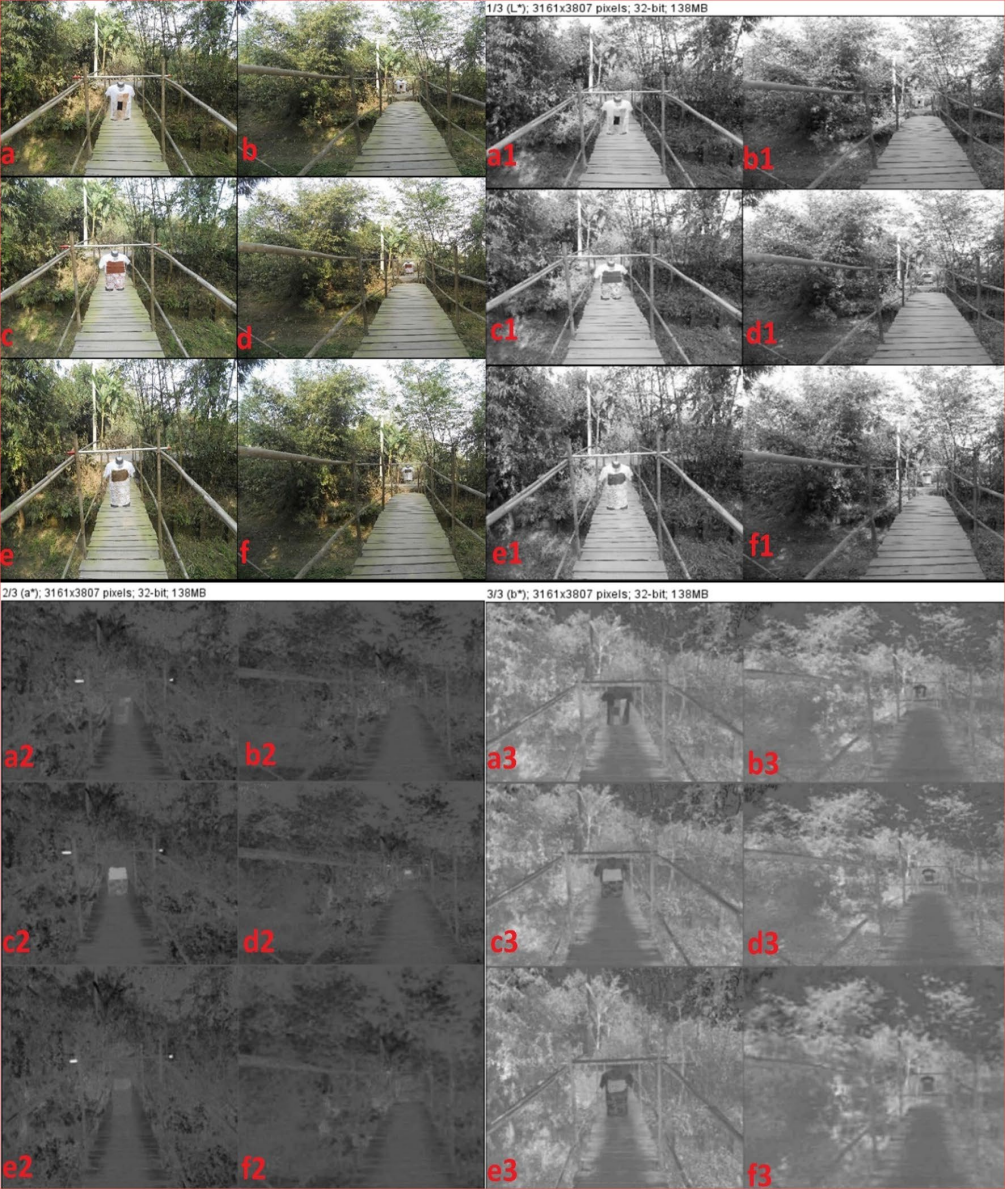
NPND-dyed-coated-printed garments placed against woodland CB and wooden bridge; and shown the photometric versus colorimetric views of L* (**a1**–**f1**), a* (**a2**–**f2**), b* (**a3**–**f3**) in grey scale imaging of RGB raw image (**a**–**f**) when the pictorial mapping has been engendered as a1 = a2 = a3 = a4; b1 = b2 = b3 = b4; c1 = c2 = c3 = c4; d1 = d2 = d3 = d4; e1 = e2 = e3 = e4; f1 = f2 = f3 = f4 for chromatic comparison.

**Figure 11:**
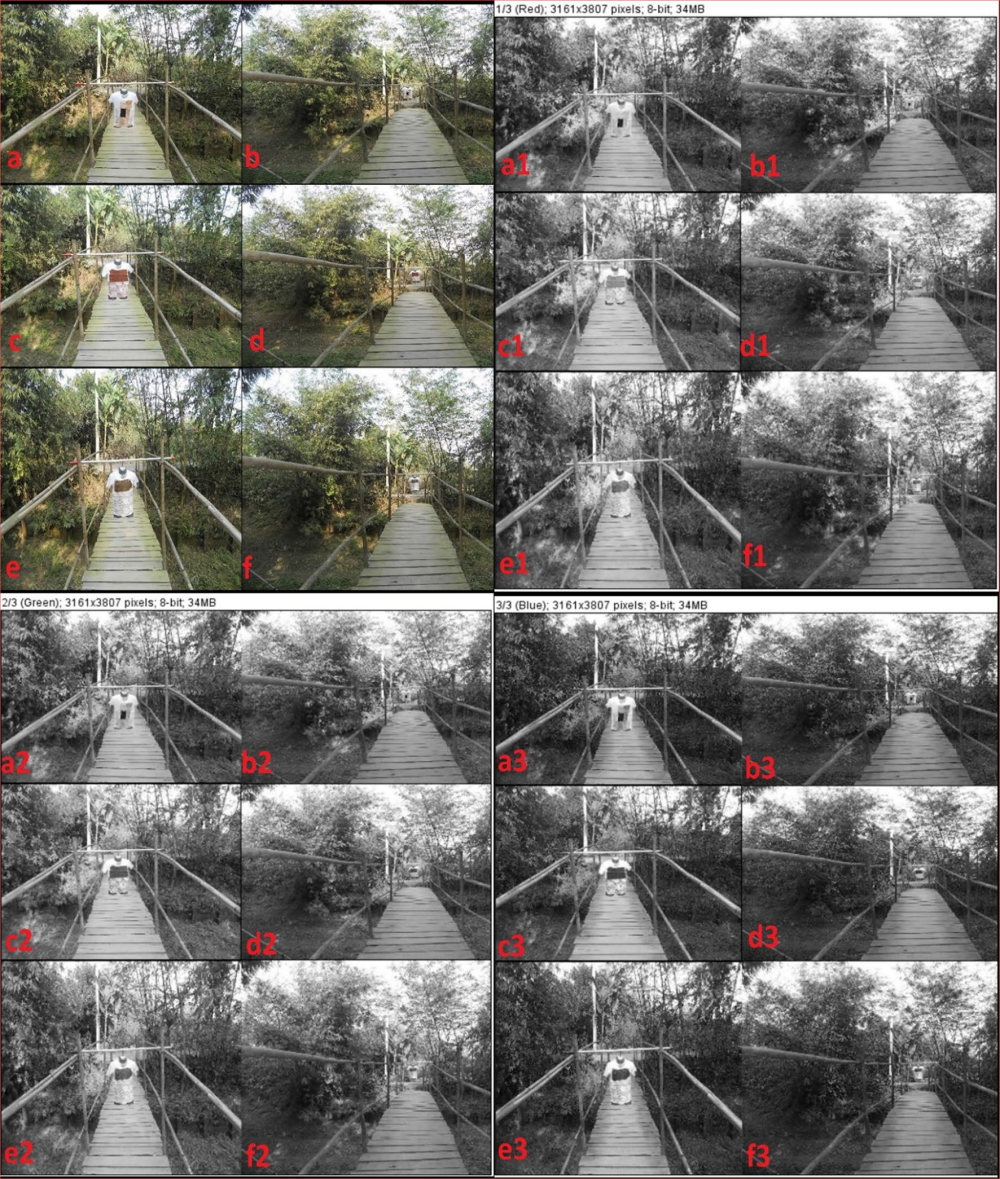
NPND-dyed-coated-printed garments placed against woodland CB and wooden bridge; and shown the photometric versus colorimetric views of red (**a1**–**f1**), green (**a2**–**f2**), blue (**a3**–**f3**) in grey scale imaging of RGB raw image (**a**–**f**) when the pictorial mapping has been engendered as a1 = a2 = a3 = a4; b1 = b2 = b3 = b4; c1 = c2 = c3 = c4; d1 = d2 = d3 = d4; e1 = e2 = e3 = e4; f1 = f2 = f3 = f4 for chromatic comparison.

Figures 10b,d,f and 11b,d,f; RGB raw image of dyed-coated-printed parts of garments have been appeared as concealed against woodland CB compared by standardized black and white. Wood chemistry of wooden bridge relates to cellulose, lignin, hemicellulose and chromatic elements of phytochemicals which have structural, compositional and chromatic similarity with dyed-coated-printed textiles with *Swietenia Macrophylla*. Similarly dry leaves and green leaves chemistry have also chromatic similarity with dyed-coated-printed textiles with NPND materials. Forest tree & wooden bridge may have variety of biological agents such as fungi, bacteria, insects which are uncontrollable parameters for chromatic hue such as blue stain, white rot and brown rot although chromatic compositions are aligned with NPND-dyed-coated-printed textiles. Figures 10a,c and 11a,c; RGB raw image of dyed-coated-printed textiles have been remarked as detected due to lacking of chlorophyl in NPND dyed-coated-printed part of garment but the target signature looks camouflage against soil background and looks detected against concrete background. Figures 10e,f and 11e,f; the chromatic hue of dry leaves of *Corchorus Capsularis* looks greenish to brownish. *Corchorus Capsularis* has existence with greenish tone, hence the coated-printed area of garment looks color matching against woodland CB of *Bamboo Vulgaris* and *Musa Acuminata*. Figures 10a1–f1; L* image of dyed-coated-printed garment looks color matching and completely concealed against a constructed wooden bridge made by *Eucalyptus Citriodora*, *Bamboo Vulgaris*; woodland CB of *Bamboo Vulgaris* and *Musa Acuminata*, soil and concrete CB; compared by standardized white and black. Figure 10a2,b2,e2,f2,a3,b3; similarly, a* & b* image of dyed-coated-printed parts of garments looks concealed and Fig. 10c2,d2,c3,d3,e3,f3; a* & b* image of coated-printed parts of garments looks detected against woodland CB. Figure 11a1–f1,a2–f2,a3–f3; under RGB image analysis, the image of dyed-coated-printed textiles looks exceptionally color matching & camouflage against a constructed wooden bridge made by *Eucalyptus Citriodora*, *Bamboo Vulgaris*; woodland CB of *Bamboo Vulgaris* and *Musa Acuminata*; soil and concrete CB; compared by standardized white and black.

### Chromatic analysis of L*, a*, b*, RGB of NPND-dyed-coated-printed garments against combat backgrounds

**Figure 12:**
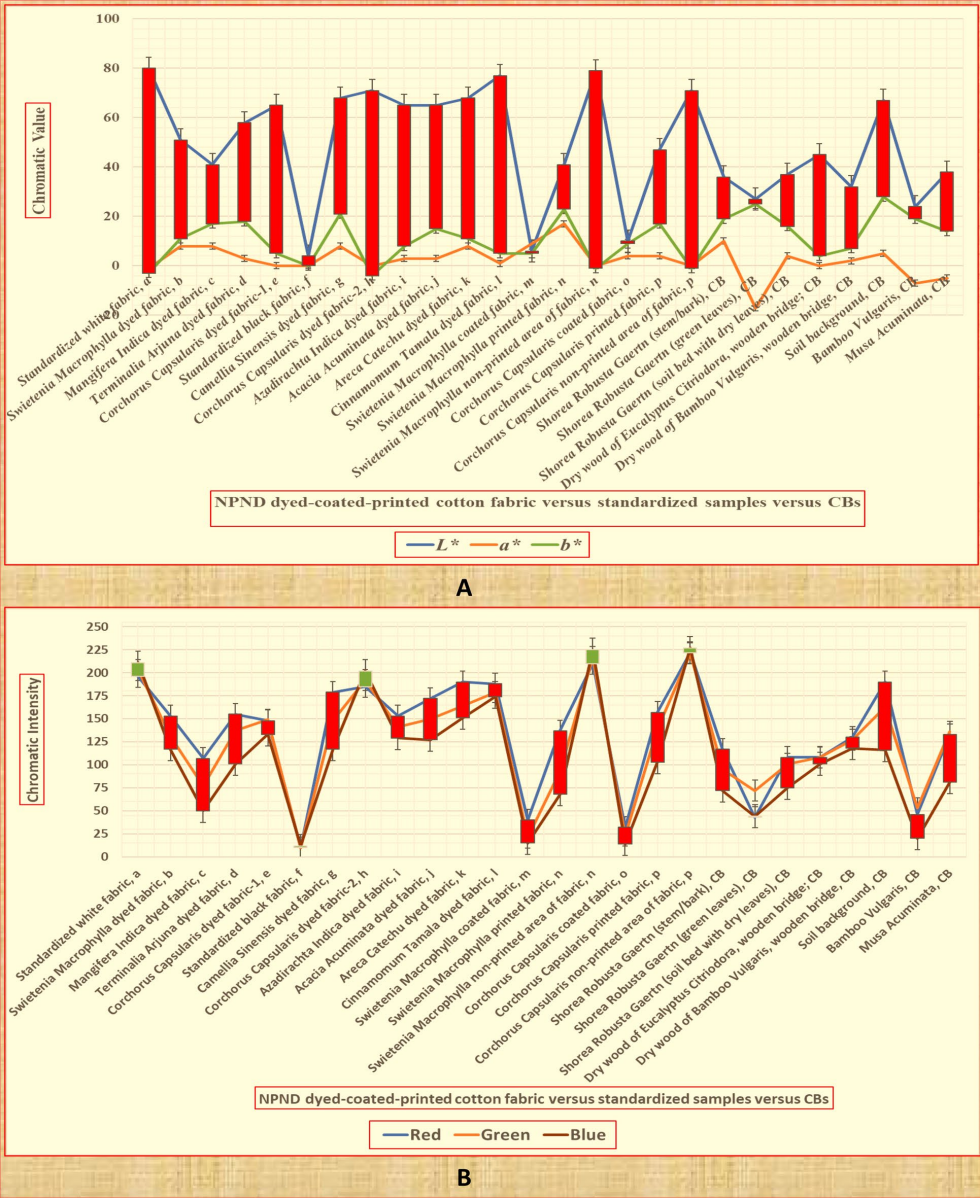
Quantitative comparison of CIE L*, a*, b* (**A**) and RGB (**B**) values for NPND dyed-coated-printed part placed against multidimensional CBs.

Figure 12A, CIE L*, a*, b* values of NPND-coated-printed textiles are more symmetrical with CB materials in general rather than NPND-dyed textiles due to chromatic matching between treated textiles and CB materials. There are also exceptions of symmetric matching of L*, a*, b* for colorful dyed fabric such as *Swietenia Macrophylla*, *Mangifera Indica*, *Terminala Arjuna*, *Camellia Sinensis* and *Acacia Acuminata*. L* value of *Corchorus Capsularis* and *Cinnamomum Tamala* are comparatively higher due to lower interactions of dye-fibre and mordant free coloration in NPND-dyebath. L*, a*, b* values of *Swietenia Macrophylla* and *Corchorus Capsularis* coated fabric are exceptionally matching against woodland CB of *Shorea Robusta Gaertn* (green leaves) and *Bamboo Vulgaris* (green leaves). L* value of *Swietenia Macrophylla* dyed fabric is comparatively lower due to NPND-mordanting of *Areca Catechu* and comparatively higher dye-fibre interactions in NPND-dyebath^18^. Figure 12B, similarly CIE trichromatic intensity of *Swietenia Macrophylla* and *Corchorus Capsularis* coated fabric are mostly matching with the selected CBs of *Shorea Robusta Gaertn* (stem/bark); *Shorea Robusta Gaertn* (green leaves); *Shorea Robusta Gaertn* (soil bed with dry leaves); Dry wood of *Eucalyptus Citriodora*, wooden bridge; Dry wood of *Bamboo Vulgaris*; wooden bridge; Soil background; *Bamboo Vulgaris* and *Musa Acuminata*. The RGB chromatic intensity of *Swietenia Macrophylla*, *Mangifera Indica*, *Terminalia Arjuna*, *Camellia Sinensis*, *Azadirachta Indica*, *Acacia Acuminata*, and *Areca Catechu* dyed fabric have been found comparatively matching with multidimensional CBs related to woodland. The chromatic data information of NPND materials and CB materials have been cited in supporting information, Table 13.

## Concluding remarks

NPND dyeing process can be replaced by coating/printing in terms of higher percentage of NPND deposition/leafy design development on fabric surface which may have more symmetry between defence target object (camouflage treated textiles) and the materials of woodland CB, this formulation may be a new model of NPND formulated camouflage textiles for concealment of target detection for defence applications in terms of ecofriendly dyes; and NPND-mordanting process for NPND based natural coloration. The phenolic and tannin reflection between *Swietenia Macrophylla* treated fabric and raw *Swietenia Macrophylla* have not been found any significant difference in terms of UV–Vis–NIR spectrums from 220 to 1400 nm which may signify a symmetrical matching of optical reflection between *Swietenia Macrophylla* treated textiles and woodland CB. The minor deviation of chromatic reflection between woodland CB, *Shorea Robusta Gaertn* and *Swietenia Macrophylla* dyed-coated-printed textiles have also been found in field trialling. *Swietenia Macrophylla* bark has been remarked as good coloration property, applicable for dyeing-coating-printing; and the reflection properties are suitable for camouflage coloration against woodland CB. Waste-NPND powder of wood processing industries may be a good source of combat-NPND. Hence, there is possibility for development of NPND treated camouflage coloration for defence protection such as defence clothing/temporary tents/nets for combat locations against woodland CB; preferred textile technologies are coating/printing for maximum deposition of NPND on textile substances. Furthermore, NPND coated/printed/dyed fabric may be used for manufacture of UV protective clothing for defence protection of simultaneous ‘camouflage textiles’ and ‘UV-protective textiles’. An ecofriendly concept of NPND coloration was applied in addition to mordant free coloration and/or NPND-mordanting/bio-mordanting without applications of any synthetic mordant. Therefore coating/printing method of NPND treated textiles have been found more efficient for camouflage formulation and coloration. NPND-dyed-coated-printed textiles can be formulated for defence clothing, protection net/tent in CB. For photographic reflection of coated and printed textiles as target object and selected woodland background as target woodland CB; coated textile is more suitable but a combination of coated-printed-NPND textiles or dyed-printed-NPND textiles can be recommended for making a leafy design on fabric against woodland CB in terms of defence clothing. NPND coated textiles is more suitable technique for symmetrical reflection with woodland CB. For example: coating of net and tent for defence protection against woodland CB. Hence, there is enormous possibilities of research on natural coloration but the concept of NPND based camouflage textiles is a new experimentation for defence applications and an ecofriendly formulation of NPND coloration. Although NPND-mordanting has been opined in this experiment but the application of synthetic mordant may enhance the functional activity of tannase for more dye-fibre crosslinking of NPND based textile coloration. Furthermore, NPND nanoparticle-synthetic binder-cotton/synthetic fabric can be applied for coated/printed fabrication against selected CB for achievement of desired camouflage textiles. Hence, NPND-natural dyes-natural mordant-natural fibre-cotton can also be implemented/extended for ecofriendly production of fashionable garments specially for production of kid’s garments/medical textiles in terms of user's friendly/protection textiles.

It is very critical issue to decide a specific NPND material for a very specific standardizing of concealment against woodland CB due to variation of chromatic chemistry by season; chromatic chemistry by combat location; chromatic chemistry by combat region, chromatic chemistry by multidimensional part of chromatic materials such as dry leaves, green leaves, barks/stems; chromatic chemistry by different species of NPND materials, chromatic chemistry by location of woodland CB against sunlight and chromatic chemistry in multidimensional spectral region in UV–Vis–IR. To avoid difficulties, the multidimensional source of NPND materials can be combined and formulated for coated/printed technique of camouflage textiles for better color matching and spectral matching in UV–Vis–IR spectrums. Under chromatic analysis, it is feasible to implement NPND based textile coloration for chromatic matching against woodland CB. *Swietenia Macrophylla*, *Mangifera Indica*, *Terminalia Arjuna*, *Camellia Sinensis*, *Azadirachta Indica*, *Acacia Acuminata*, *Areca Catechu*, *Cinnamomum Tamala*, *Corchorus Capsularis*, *Shorea Robusta Gaertn*, *Eucalyptus Citriodora*, *Bamboo Vulgaris*, *Musa Acuminata* and related species of plants are remarked as NPND materials. Different types of NPND materials, different parts of tree (leaves, wood, bark, root, fruit peel, fruit seed, flower, etc.) and different source of waste NPND materials can be collected, grinded and mixed in textile processing industry as nano particle for even textile dyeing/coating/printing of ‘green coloration’, ‘green mordanting’ and ‘green processing’ in terms of technical property rather than coloration only. Both NPND solid coloration and NPND patterning can be considered for camouflage formulation as per combat environment and/or requirement of defence professional/region related to woodland CB.

## Supplementary Information

Supplementary Tables.

## Data Availability

All data generated or analysed during this experimentation are included in supplementary information file; Tables 1–13, supporting information.
