# Supplementary material for: UV–Visible–NIR camouflage textiles with natural plant based natural dyes on natural fibre against woodland combat background for defence protection
Source: Sci Rep. 2023 Mar 28;13:5021. doi: 10.1038/s41598-023-31725-2 (PMC10050389; doi:10.1038/s41598-023-31725-2)
Supplement: Supplementary file 1 — Supplementary Tables. [file 41598_2023_31725_MOESM1_ESM.pdf]

Supporting information

UV-Visible-NIR Camouflage Textiles with Natural Plant Based Natural Dyes on Natural Fibre against Woodland Combat Background for Defence Protection

Md. Anowar Hossain
School of Fashion and Textiles, RMIT University
25 Dawson Street, Brunswick, Melbourne, VIC 3056, Australia
(engr.anowar@yahoo.com)

Table 1. Supporting information of reflection (%) from 220 nm to 299 nm for standardized barium Sulphate, *Swietenia Macrophylla* dyed fabric without mordanting, *Swietenia Macrophylla* dyed fabric with mordanting, undyed knitted fabric, raw *Swietenia Macrophylla* and raw *Areca Catechu*

Wavelength (nm)	Standardized Barium Sulphate	Swietenia Macrophylla dyed fabric without mordanting	Swietenia Macrophylla dyed fabric with mordanting	Undyed knitted fabric	Swietenia Macrophylla	Raw Areca Catechu
220	99.8	32.168	19.7	228.989	3.028	2.782
221	100.579	31.657	18.286	221.441	3.143	2.562
222	100.221	31.226	19.243	223.061	3.283	2.398
223	99.773	30.178	18.141	222.966	3.228	2.391
224	98.709	29.926	18.033	213.562	3.801	2.435
225	98.863	29.968	18.385	211.797	3.227	2.499
226	99.453	29.954	18.178	212.969	3.496	2.541
227	100.783	30.456	18.836	218.992	3.33	2.655
228	102.328	30.952	18.984	224.049	3.587	2.475
229	101.264	30.996	19.319	220.881	3.364	2.68
230	99.393	30.86	19.134	219.081	3.148	2.531
231	101.085	31.592	18.895	222.408	3.08	2.727
232	99.37	31.892	19.505	222.137	3.306	2.42
233	99.927	33.557	20.021	227.398	3.314	2.422
234	101.446	33.415	20.816	227.929	3.022	2.539
235	101.298	34.517	20.786	233.044	3.375	2.475
236	100.877	34.694	21.182	225.271	3.171	2.325
237	101.366	35.601	21.739	231.274	3.069	2.429
238	100.053	36.872	22.114	227.112	3.23	2.39
239	99.552	36.484	22.144	226.209	3.214	2.385
240	100.513	36.263	22.28	217.807	3.285	2.529
241	101.26	35.899	22.799	215.667	3.162	2.545
242	100.306	36.01	23.099	211.186	3.367	2.451
243	99.349	35.657	22.134	203.697	3.228	2.511
244	100.368	35.798	22.682	200.299	3.333	2.514
245	99.356	35.315	22.965	190.263	3.2	2.367
246	100.481	35.094	22.464	184.195	3.163	2.349
247	99.078	33.929	22.162	176.687	2.91	2.394
248	100.996	34.457	22.465	173.921	3.227	2.476
249	99.744	33.179	22.362	165.006	3.067	2.406
250	100.227	32.899	22.542	159.895	3.128	2.474
251	100.113	32.732	22.317	156.014	3.093	2.391
252	99.461	32.027	21.817	150.213	3.112	2.25
253	100.379	31.943	22.187	145.406	3.124	2.282
254	100.3	31.167	21.584	142.042	3.164	2.328
255	100.27	30.746	21.647	136.127	3.208	2.306
256	101.664	30.54	21.441	134.467	3.151	2.363
257	99.414	30.053	21.342	130.69	2.973	2.313
258	99.693	30.287	21.164	127.834	3.128	2.354
259	100.935	29.359	20.653	124.803	3.052	2.203
260	100.584	29.322	20.617	123.178	2.948	2.23
261	100.892	29.57	21.004	123.027	2.913	2.286
262	100.347	28.994	21.311	121.519	2.949	2.315
263	100.302	29.248	20.511	121.343	2.88	2.288
264	100.691	28.86	20.626	119.663	2.87	2.188
265	98.921	28.454	20.291	117.955	2.935	2.16
266	100.334	28.688	19.861	118.038	2.852	2.111
267	101.06	28.482	20.053	118.161	2.743	2.09
268	100.77	28.31	19.75	118.767	2.607	2.109
269	99.496	27.967	19.083	117.049	2.756	2.112
270	100.615	27.702	19.433	118.108	2.703	2.206
271	100.343	27.354	18.976	117.31	2.712	2.169
272	100.576	26.945	18.694	117.041	2.684	2.097
273	100.643	26.882	18.648	117.95	2.626	2.199
274	99.069	26.357	17.983	115.849	2.605	2.043
275	100.769	26.519	18.216	115.811	2.648	2.162
276	99.905	26.205	18.125	115.734	2.672	2.163
277	100.105	25.64	17.505	114.183	2.686	2.03
278	100.487	25.267	17.443	112.787	2.646	2.059
279	100.533	25.525	17.104	111.228	2.582	2.08
280	99.747	24.712	16.892	110.196	2.757	2.136
281	99.693	24.907	17.011	108.754	2.662	2.116
282	99.575	24.428	16.852	108.06	2.775	2.124
283	100.694	23.897	16.401	107.614	2.593	2.128
284	99.782	23.769	16.41	104.913	2.711	2.103
285	100.283	23.707	16.317	103.014	2.702	2.071
286	100.339	24.049	16.436	101.623	2.8	2.109
287	100.414	23.25	16.079	98.371	2.802	2.125
288	100.452	22.885	16.163	95.455	2.623	2.241
289	100.255	22.657	16.281	93.347	2.814	2.175
290	99.908	22.672	16.106	90.86	2.742	2.236
291	100.595	22.917	16.587	87.498	2.895	2.153
292	102.044	22.783	16.859	87.566	2.921	2.283
293	100.531	23.316	16.949	85.732	2.952	2.125
294	99.849	22.749	16.752	82.808	2.838	2.266
295	99.591	23.291	17.015	82.579	3.112	2.28
296	101.199	23.33	17.684	82.541	3.115	2.206
297	99.525	23.861	17.555	80.891	3.063	2.343
298	100.597	23.469	17.404	80.93	3.149	2.26
299	99.674	23.267	17.569	80.065	3.205	2.235
		**Anowar_SI_NPND_1_RMIT**				

Open Researcher and Contributor ID (ORCID) Md. Anowar Hossain: https://orcid.org/0000-0003-2880-6287

Table 2. Supporting information of reflection (%) from 300 nm to 399 nm for standardized barium sulphate, *Swietenia Macrophylla* dyed fabric without mordanting, *Swietenia Macrophylla* dyed fabric with mordanting, undyed knitted fabric, raw *Swietenia Macrophylla* and raw *Areca Catechu*

Wavelength (nm)	Standardized Barium Sulphate	Swietenia Macrophylla dyed fabric without mordanting	Swietenia Macrophylla dyed fabric with mordanting	Undyed knitted fabric	Swietenia Macrophylla	Raw Areca Catechu
300	99.748	23.809	17.58	79.523	3.123	2.217
301	99.836	23.715	18.001	79.191	3.245	2.22
302	99.841	24.306	18.122	78.955	3.268	2.252
303	100.011	23.815	18.155	78.738	3.346	2.224
304	101.983	25.146	18.641	79.686	3.282	2.286
305	100.654	24.77	18.917	78.553	3.442	2.351
306	99.58	24.339	18.43	78.918	3.425	2.27
307	100.681	24.964	18.607	77.737	3.362	2.538
308	99.46	26.206	18.801	79.909	3.387	2.476
309	98.465	25.531	19.182	76.691	3.479	2.272
310	99.326	25.239	20.144	77.577	3.537	2.333
311	100.237	25.952	19.792	78.87	3.86	2.676
312	101.78	26.614	19.454	77.484	3.486	2.241
313	99.409	25.501	19.547	77.407	3.608	2.402
314	102.339	25.558	20.559	78.397	3.225	2.221
315	99.67	25.177	19.539	79.071	3.751	2.436
316	101.337	25.92	20.23	79.222	3.408	2.208
317	99.096	26.021	20.221	78.678	3.485	2.337
318	100.522	27.051	20.391	78.613	3.323	2.278
319	101.566	25.934	20.67	77.658	4.003	2.56
320	101.065	27.017	21.204	81.138	3.755	2.275
321	97.99	26.67	21.147	78.064	4.261	2.474
322	99.273	27.109	21.796	79.693	4.118	2.776
323	102.365	27.335	20.563	78.364	4.047	2.319
324	99.861	28.229	22.372	79.329	3.784	2.425
325	100.381	26.88	21.556	77.547	3.972	2.421
326	100.406	27.909	21.881	77.31	4.215	2.328
327	99.519	27.252	21.478	77.213	4.102	2.507
328	99.536	27.623	21.919	78.014	4.092	2.467
329	99.274	27.805	22.023	79.04	4.408	2.532
330	99.564	27.304	22.438	76.864	3.934	2.411
331	101.474	27.705	22.386	79.071	4.26	2.669
332	100.169	28.047	22.51	78.151	4.317	2.749
333	100.282	27.927	22.297	79.098	4.466	2.734
334	99.546	27.624	22.012	79.031	4.321	2.56
335	101.428	28.032	22.602	78.105	4.557	2.319
336	101.956	27.044	22.045	76.574	4.665	2.61
337	98.439	28.359	21.67	76.954	4.528	2.489
338	99.58	27.617	23.147	77.034	4.399	2.702
339	100.136	27.716	22.568	78.12	4.573	2.57
340	99.47	28.222	22.49	78.093	4.571	2.648
341	102.172	27.936	22.625	76.603	4.745	2.616
342	100.145	27.315	22.483	76.841	4.929	2.725
343	99.335	27.374	22.598	76.493	4.754	2.541
344	101.764	28.035	23.041	77.461	4.913	2.706
345	99.87	27.61	22.792	77.107	4.847	2.713
346	100.349	28.684	22.962	77.186	5.151	2.824
347	100.622	27.674	22.775	76.633	4.832	2.613
348	99.424	27.754	23.172	75.722	5.036	2.533
349	99.311	27.979	23.025	75.581	4.96	2.755
350	100.115	27.911	23.31	76.347	5.303	2.806
351	101.012	27.64	23.185	76.632	5.381	2.699
352	100.009	27.953	22.915	75.309	5.626	2.602
353	100.127	27.939	22.146	75.155	5.461	2.821
354	100.93	27.92	23.04	74.381	5.637	2.789
355	100.42	27.959	23.391	75.073	5.558	2.614
356	99.635	27.315	22.47	73.553	5.655	2.695
357	98.744	27.444	22.081	73.949	5.669	2.804
358	101.72	27.669	23.007	74.099	5.74	2.74
359	100.299	27.386	23.295	73.747	5.938	2.515
360	100.995	27.624	22.923	73.426	6.398	2.766
361	102.01	27.512	22.693	73.909	6.225	2.745
362	99.887	27.308	22.328	73.27	6.169	2.955
363	99.871	27.271	22.655	72.802	6.085	2.809
364	100.315	27.436	23.351	73.301	6.147	3.007
365	98.319	27.009	22.769	71.307	6.389	2.839
366	98.546	26.697	22.447	70.321	6.453	2.883
367	100.506	27.542	22.738	71.902	6.771	2.929
368	102.21	26.589	22.514	70.784	7.101	3.106
369	99.314	27.403	22.393	71.444	7.241	3.109
370	100.008	27.293	22.879	71.691	7.059	2.956
371	99.404	27.188	23.403	71.36	7.209	3.136
372	99.44	26.539	23.055	70.076	7.23	3.013
373	99.917	27.241	22.544	71.366	7.356	2.953
374	100.887	27.012	22.918	71.429	7.454	3.055
375	99.66	27.167	22.65	70.92	7.51	3.098
376	100.103	27.052	22.765	71.096	7.845	3.078
377	99.703	27.105	22.682	70.461	7.877	3.05
378	99.548	27.036	22.882	70.739	8.045	3.051
379	100.067	27.06	22.65	70.372	8.052	3.052
380	100.309	27.243	22.801	70.796	8.343	3.056
381	100.893	27.104	22.831	70.485	8.435	3.088
382	99.943	27.217	22.776	70.615	8.565	3.159
383	100.044	27.106	22.945	70.545	8.731	3.156
384	100.162	27.178	23.162	70.37	8.843	3.169
385	99.948	27.227	23.121	70.154	9.089	3.196
386	100.286	27.402	23.226	70.322	9.133	3.203
387	99.427	27.359	23.078	70.035	9.218	3.178
388	100.175	27.348	23.126	69.896	9.358	3.254
389	100.457	27.523	23.472	70.073	9.649	3.327
390	99.961	27.536	23.488	70.028	9.719	3.231
391	100.274	27.839	23.672	70.329	9.728	3.288
392	100.605	27.795	23.705	70.464	10.006	3.256
393	100.062	27.692	23.68	70.021	10.044	3.249
394	100.245	27.901	23.899	70.179	10.281	3.288
395	99.822	28.002	23.832	70.372	10.364	3.282
396	99.85	28.099	23.991	70.107	10.404	3.322
397	100.165	27.913	24.068	70.834	10.625	3.334
398	100.04	27.969	24.077	70.244	10.728	3.378
399	100.118	28.302	24.213	70.697	10.817	3.353
		**Anowar_SI_NPND_2_RMIT**				

Open Researcher and Contributor ID (ORCID) Md. Anowar Hossain: https://orcid.org/0000-0003-2880-6287

Table 3. Supporting information of reflection (%) from 400 nm to 499 nm for standardized barium sulphate, *Swietenia Macrophylla* dyed fabric without mordanting, *Swietenia Macrophylla* dyed fabric with mordanting, undyed knitted fabric, raw *Swietenia Macrophylla* and raw *Areca Catechu*.

Wavelength (nm)	Standardized Barium Sulphate	Swietenia Macrophylla dyed fabric without mordanting	Swietenia Macrophylla dyed fabric with mordanting	Undyed knitted fabric	Swietenia Macrophylla	Raw Areca Catechu
400	100.274	28.307	24.186	71.01	10.967	3.384
401	100.214	28.32	24.251	70.984	10.982	3.404
402	100.022	28.479	24.369	71.247	11.174	3.371
403	100.296	28.635	24.509	71.03	11.212	3.426
404	99.989	28.725	24.688	70.819	11.278	3.412
405	100.416	28.931	24.871	71.397	11.441	3.412
406	99.976	29.015	24.878	71.466	11.385	3.502
407	99.747	29.28	25.319	71.674	11.542	3.457
408	100.155	29.538	25.51	71.894	11.631	3.523
409	99.677	29.711	25.608	71.922	11.629	3.477
410	100.674	30.104	25.926	72.826	11.702	3.501
411	99.84	30.301	26.027	72.598	11.684	3.527
412	100.419	30.638	26.357	73.309	11.921	3.542
413	100.141	30.753	26.647	73.336	11.915	3.537
414	100.036	31.02	26.711	73.89	11.902	3.577
415	100.601	31.406	26.95	74.235	11.979	3.635
416	100.065	31.44	27.143	74.406	12.104	3.562
417	100.58	31.859	27.424	74.93	12.097	3.575
418	100.525	32.038	27.474	75.415	12.141	3.652
419	100.457	32.214	27.719	75.782	12.097	3.639
420	100.08	32.153	27.781	75.711	12.102	3.663
421	99.847	32.407	27.904	76.468	12.017	3.641
422	100.42	32.428	28.011	76.729	12.245	3.642
423	100.224	32.404	28.027	76.744	12.13	3.664
424	100.56	32.703	28.323	77.455	12.227	3.715
425	100.133	32.38	28.027	77.22	12.198	3.681
426	100.162	32.574	28.165	77.763	12.25	3.709
427	99.588	32.481	28.053	77.655	12.209	3.679
428	100.206	32.486	28.232	77.794	12.186	3.723
429	100.543	32.728	28.359	78.274	12.308	3.756
430	100.188	32.576	28.214	78.361	12.29	3.722
431	99.813	32.5	28.03	78.228	12.171	3.686
432	99.91	32.588	28.31	78.572	12.239	3.808
433	100.151	32.467	28.046	78.502	12.307	3.774
434	100.689	32.612	28.259	78.656	12.27	3.809
435	100.252	32.697	28.319	79.141	12.208	3.796
436	100.138	32.475	28.112	78.952	12.136	3.78
437	100.056	32.419	28.056	78.522	12.311	3.743
438	100.263	32.42	28.084	78.93	12.237	3.816
439	100.27	32.492	28.203	79.057	12.206	3.854
440	99.742	32.453	28.016	79.338	12.161	3.842
441	100.021	32.429	28.122	79.178	12.261	3.79
442	100.027	32.232	28.024	79.366	12.06	3.852
443	100.383	32.231	27.935	79.108	12.25	3.888
444	100.154	32.126	28.027	79.05	12.184	3.832
445	99.996	32.09	27.897	79.547	12.236	3.864
446	100.282	32.14	27.894	79.167	12.205	3.859
447	100.195	32.013	27.806	79.282	12.084	3.885
448	99.796	31.984	27.866	79.229	12.074	3.889
449	100.403	32.062	28.075	79.878	12.097	3.922
450	100.157	32.158	27.954	79.902	12.052	3.894
451	99.735	32.062	27.861	79.378	12.163	3.906
452	100.137	32.074	27.943	79.641	12.053	3.915
453	99.832	32.237	27.959	79.654	12.081	3.967
454	100.172	32.175	27.78	79.86	12.13	3.94
455	100.156	32.208	27.94	79.958	12.197	3.935
456	100.205	32.263	27.736	79.872	12.124	3.959
457	100.41	32.317	27.929	80.003	12.156	4.01
458	100.261	32.396	27.967	80.018	12.14	3.997
459	100.334	32.401	28.042	79.821	12.226	4.025
460	100.337	32.445	28.079	80.129	12.315	4.011
461	100.646	32.465	28.133	80.324	12.315	4.085
462	100.009	32.458	27.971	79.908	12.169	4.064
463	100.309	32.666	28.119	80.133	12.267	4.055
464	100.262	32.606	28.1	79.988	12.298	4.067
465	100.089	32.497	28.015	80.145	12.303	4.069
466	100.172	32.648	28.346	80.4	12.371	4.074
467	100.338	32.715	28.428	80.676	12.375	4.139
468	100.36	32.796	28.285	80.101	12.41	4.168
469	100.19	32.706	28.3	80.353	12.446	4.127
470	100.087	32.652	28.198	80.128	12.366	4.155
471	100.327	32.843	28.347	80.38	12.482	4.18
472	100.595	32.74	28.432	80.398	12.577	4.229
473	99.895	32.556	28.262	80.003	12.542	4.186
474	100.027	32.752	28.301	80.314	12.589	4.203
475	100.288	32.674	28.306	80.279	12.558	4.255
476	100.274	32.627	28.411	80.465	12.615	4.257
477	100.118	32.688	28.267	80.347	12.699	4.302
478	100.336	32.886	28.354	80.762	12.743	4.261
479	100.39	32.597	28.309	80.617	12.781	4.313
480	99.872	32.604	28.316	80.268	12.811	4.338
481	99.996	32.474	28.303	80.216	12.859	4.322
482	100.371	32.72	28.39	80.384	13.011	4.298
483	100.27	32.727	28.493	80.511	13.054	4.379
484	99.888	32.675	28.346	80.423	13.048	4.373
485	100.406	32.901	28.666	80.688	13.221	4.417
486	100.129	32.897	28.692	80.55	13.306	4.42
487	100.344	32.85	28.819	80.566	13.409	4.455
488	99.876	32.89	28.705	80.194	13.42	4.422
489	100.279	33.299	28.929	80.723	13.617	4.526
490	100.085	33.311	28.933	80.579	13.714	4.534
491	99.936	33.334	29.064	80.442	13.732	4.515
492	100.285	33.574	29.152	80.818	14.02	4.567
493	100.2	33.685	29.348	80.717	14.131	4.624
494	99.915	33.739	29.345	80.696	14.23	4.62
495	100.134	33.902	29.521	80.856	14.457	4.61
496	100.416	34.131	29.718	80.986	14.609	4.665
497	100.673	34.18	29.935	81.155	14.789	4.757
498	100.112	34.325	29.914	80.944	14.972	4.707
499	99.829	34.428	30.099	80.727	15.099	4.796
		**Anowar_SI_NPND_3_RMIT**				

Open Researcher and Contributor ID (ORCID) Md. Anowar Hossain: https://orcid.org/0000-0003-2880-6287

Table 4. Supporting information of reflection (%) from 500 nm to 599 nm for standardized barium sulphate, *Swietenia Macrophylla* dyed fabric without mordanting, *Swietenia Macrophylla* dyed fabric with mordanting, undyed knitted fabric, raw *Swietenia Macrophylla* and raw *Areca Catechu*.

Wavelength (nm)	Standardized Barium Sulphate	Swietenia Macrophylla dyed fabric without mordanting	Swietenia Macrophylla dyed fabric with mordanting	Undyed knitted fabric	Swietenia Macrophylla	Raw Areca Catechu
500	100.509	34.583	30.341	80.806	15.256	4.808
501	100.085	34.708	30.252	80.79	15.383	4.824
502	100.168	34.773	30.395	80.962	15.637	4.906
503	100.353	35.029	30.744	80.877	15.824	4.923
504	100.058	35.119	30.626	80.932	16.051	4.931
505	100.002	35.218	30.897	81.158	16.242	5.01
506	100.49	35.408	31.02	80.943	16.385	5.033
507	100.345	35.509	31.11	81.015	16.601	5.084
508	100.535	35.699	31.295	81.195	16.809	5.092
509	100.222	35.735	31.439	81.015	17.057	5.138
510	100.296	35.928	31.519	81.068	17.208	5.206
511	100.184	35.973	31.625	80.926	17.393	5.212
512	100.037	36.033	31.803	80.844	17.49	5.289
513	99.946	36.223	31.813	80.37	17.793	5.294
514	100.194	36.498	31.968	80.999	18.025	5.381
515	100.177	36.427	32.2	80.892	18.272	5.405
516	100.037	36.661	32.165	80.713	18.317	5.484
517	100.439	36.713	32.465	80.982	18.682	5.509
518	100.163	36.9	32.517	80.97	18.879	5.596
519	99.901	37.101	32.741	81.048	19.138	5.604
520	99.951	37.248	32.832	81.027	19.348	5.617
521	100.455	37.511	33.146	81.229	19.635	5.672
522	100.262	37.53	33.139	81.054	19.857	5.74
523	100.017	37.777	33.338	80.875	19.815	5.847
524	100.023	37.63	33.454	80.429	20.102	5.818
525	100.037	38.049	33.619	81.183	20.37	5.866
526	100.077	38.257	33.695	80.607	20.647	5.934
527	100.091	38.388	33.869	80.926	20.784	6.063
528	100.357	38.613	34.257	80.954	21.053	6.049
529	99.906	38.623	34.296	80.855	21.262	6.117
530	100.157	39.017	34.453	80.889	21.51	6.181
531	99.742	39.092	34.584	80.776	21.705	6.188
532	100.36	39.627	34.8	81.231	21.932	6.271
533	100.033	39.463	35.112	80.87	22.185	6.32
534	100.279	39.982	35.263	81.002	22.378	6.411
535	99.838	40.121	35.475	81.016	22.498	6.501
536	100.431	40.62	35.613	81.262	22.889	6.524
537	100.464	40.842	35.774	81.136	23.128	6.575
538	100.153	40.994	36.078	81.076	23.303	6.635
539	99.764	41.179	36.308	80.766	23.38	6.705
540	100.153	41.359	36.484	81.192	23.689	6.729
541	100.194	41.658	36.769	80.865	23.95	6.754
542	100.175	41.808	36.725	81.017	24.08	6.868
543	100.119	42.101	37.105	81.033	24.388	6.902
544	100.042	42.305	37.332	80.954	24.558	6.949
545	100.406	42.632	37.507	81.256	24.806	6.994
546	99.901	42.845	37.643	80.973	24.925	7.061
547	99.978	43.171	37.939	80.758	25.054	7.096
548	100.423	43.457	38.173	81.129	25.405	7.18
549	99.857	43.373	38.127	80.735	25.433	7.189
550	100.163	44.034	38.572	81.049	25.838	7.344
551	99.94	44.053	38.726	80.98	26.012	7.383
552	100.236	44.525	39.044	81.109	26.249	7.467
553	100.402	44.613	39.242	81.059	26.487	7.536
554	100.279	44.787	39.377	80.974	26.677	7.502
555	99.846	45.2	39.444	81.094	26.681	7.593
556	100.202	45.351	39.75	80.89	26.947	7.665
557	100.021	45.546	40.008	81.056	27.189	7.698
558	100.245	45.974	40.231	81.269	27.434	7.741
559	100.421	46.227	40.513	81	27.729	7.841
560	100.048	46.387	40.537	81.069	27.866	7.882
561	100.161	46.693	40.885	80.989	28.019	7.893
562	100.264	46.901	41.081	80.937	28.254	8.022
563	100.116	47.201	41.274	80.943	28.365	8.066
564	100.621	47.568	41.524	81.164	28.604	8.182
565	100.398	47.723	41.766	81.179	28.72	8.186
566	100.448	47.862	41.817	81.27	28.933	8.205
567	100.342	48.143	42.043	81.058	29.068	8.285
568	100.506	48.657	42.353	81.19	29.381	8.365
569	100.027	48.586	42.385	81.01	29.378	8.36
570	100.143	48.876	42.78	81.147	29.648	8.488
571	100.401	49.1	42.876	81.311	29.948	8.517
572	100.106	49.384	43.016	81.059	30.001	8.575
573	99.955	49.476	43.209	81.028	30.264	8.642
574	100.004	49.888	43.497	81.332	30.453	8.654
575	100.067	50.005	43.631	81.397	30.528	8.748
576	100.347	50.418	43.916	81.544	30.864	8.837
577	100.107	50.385	44.128	81.403	30.959	8.886
578	100.053	50.73	44.265	81.353	31.227	8.923
579	100.395	51.015	44.393	81.569	31.447	9.001
580	100.142	51.279	44.678	81.452	31.654	9.132
581	99.97	51.272	44.952	81.164	31.849	9.124
582	100.189	51.7	45.178	81.498	32.07	9.253
583	100.032	51.96	45.262	81.535	32.256	9.281
584	100.232	52.117	45.527	81.383	32.514	9.289
585	99.875	52.22	45.66	81.345	32.855	9.413
586	99.87	52.476	45.782	81.311	32.987	9.538
587	100.201	52.791	46.068	81.6	33.37	9.567
588	100.371	53.039	46.332	81.609	33.635	9.713
589	100.227	53.201	46.422	81.42	33.856	9.724
590	100.104	53.356	46.637	81.554	34.254	9.834
591	100.167	53.674	46.885	81.577	34.527	9.888
592	100.317	53.831	46.982	81.676	34.88	10.018
593	100.04	54.067	47.183	81.584	35.112	10.044
594	100.265	54.055	47.45	81.576	35.48	10.15
595	100.174	54.49	47.56	81.535	35.912	10.284
596	100.377	54.583	47.665	81.36	36.178	10.331
597	100.406	54.83	48.005	81.592	36.673	10.421
598	100.366	55.184	48.289	81.433	36.894	10.519
599	100.222	55.448	48.451	81.563	37.491	10.617
		**Anowar_SI_NPND_4_RMIT**				

Open Researcher and Contributor ID (ORCID) Md. Anowar Hossain: https://orcid.org/0000-0003-2880-6287

Table 5. Supporting information of reflection (%) from 600 nm to 699 nm for standardized barium sulphate, *Swietenia Macrophylla* dyed fabric without mordanting, *Swietenia Macrophylla* dyed fabric with mordanting, undyed knitted fabric, raw *Swietenia Macrophylla* and raw *Areca Catechu*.

Wavelength (nm)	Standardized Barium Sulphate	Swietenia Macrophylla dyed fabric without mordanting	Swietenia Macrophylla dyed fabric with mordanting	Undyed knitted fabric	Swietenia Macrophylla	Raw Areca Catechu
600	100.417	55.762	48.653	81.677	37.863	10.794
601	100.113	55.804	48.795	81.663	38.292	10.866
602	100.235	56.009	49.151	81.623	38.871	10.851
603	100.154	55.969	49.109	81.352	39.02	10.98
604	99.976	56.421	49.24	81.485	39.589	11.083
605	100.041	56.633	49.627	81.467	39.989	11.24
606	100.252	56.775	49.687	81.604	40.621	11.352
607	100.222	57.103	50.163	81.623	40.926	11.427
608	100.258	57.402	50.421	81.62	41.494	11.645
609	100.322	57.598	50.488	81.66	42.001	11.624
610	100.18	57.922	50.544	81.821	42.371	11.883
611	100.092	58.026	50.748	81.392	42.705	11.839
612	100.009	58.046	50.895	81.558	43.157	12.088
613	100.278	58.379	51.231	82.079	43.936	12.162
614	100.287	58.735	51.437	81.726	44.129	12.263
615	99.938	58.856	51.474	81.814	44.628	12.332
616	100.144	59.247	51.779	82.175	45.234	12.55
617	100.067	59.321	51.984	81.948	45.445	12.641
618	99.897	59.439	52.21	81.942	46.062	12.709
619	100.165	59.739	52.365	82.116	46.457	12.827
620	100.27	59.844	52.543	82.216	46.697	12.996
621	100.053	60.166	52.81	82.277	47.239	13.123
622	100.226	60.279	53.111	82.266	47.598	13.319
623	100.116	60.376	53.094	82.329	47.791	13.388
624	100.237	60.906	53.477	82.617	48.43	13.463
625	99.88	61.015	53.7	82.305	48.881	13.569
626	100.197	61.22	54.026	82.678	49.263	13.76
627	100.295	61.614	54.129	82.491	49.52	13.864
628	100.253	61.672	54.397	82.686	49.939	14
629	100.105	61.889	54.426	82.606	50.307	14.128
630	100.083	61.967	54.545	82.747	50.702	14.245
631	99.929	62.397	54.832	82.644	51.02	14.305
632	100.308	62.509	55.019	82.838	51.393	14.49
633	100.034	62.518	55.104	82.671	51.604	14.549
634	100.058	62.583	55.47	82.715	52.028	14.702
635	100.095	62.917	55.592	82.799	52.237	14.842
636	100.352	62.876	55.874	82.754	52.788	14.957
637	100.355	63.436	56.088	82.643	52.981	15.089
638	99.725	63.233	56.023	82.596	53.165	15.125
639	100.029	63.748	56.377	82.858	53.579	15.208
640	100.116	63.691	56.447	82.962	53.953	15.467
641	100.372	64.109	56.612	82.951	54.167	15.463
642	100.319	64.101	56.925	83.097	54.468	15.689
643	100.003	64.417	57.04	83.007	54.754	15.74
644	100.144	64.51	57.268	82.959	55.016	15.916
645	99.786	64.413	57.445	82.922	55.256	15.952
646	100.015	64.914	57.667	83.081	55.679	16.146
647	100.215	64.973	57.647	82.912	55.881	16.158
648	100.447	65.504	57.921	83.359	56.429	16.382
649	100.226	65.423	58.139	83.071	56.291	16.455
650	100.223	65.788	58.365	83.005	57.005	16.667
651	100.293	65.989	58.429	83.355	57.25	16.821
652	100.147	66.037	58.669	83.281	57.682	16.855
653	100.122	66.147	58.645	83.232	57.855	16.976
654	100.342	66.029	58.988	83.12	57.924	17.195
655	100.336	66.304	59.248	83.438	58.294	17.298
656	100.478	66.745	59.519	83.229	58.704	17.414
657	100.019	66.683	59.406	83.054	58.636	17.474
658	100.02	66.715	59.475	83.088	59.069	17.703
659	100.18	66.944	59.763	83.083	58.965	17.721
660	100.077	67.092	59.928	83.324	59.561	17.914
661	100.269	67.072	59.944	82.691	59.833	17.979
662	100.222	67.281	60.344	83.217	59.891	17.973
663	100.289	67.541	60.512	83.513	60.457	18.256
664	100.153	67.67	60.65	83.183	60.747	18.352
665	100.113	68.025	60.843	83.225	60.507	18.488
666	100.63	68.3	61.227	83.686	61.314	18.744
667	100.488	68.317	60.907	83.241	61.529	18.796
668	100.378	68.337	61.229	83.394	61.576	18.885
669	100.157	68.359	61.413	83.503	61.717	19.086
670	100.294	68.576	61.607	83.472	62.066	19.129
671	100.341	68.947	61.776	83.486	62.29	19.307
672	100.539	69.081	62.062	83.66	62.499	19.401
673	99.834	68.688	61.729	82.772	62.603	19.447
674	99.812	69.348	62.04	83.516	62.892	19.667
675	100.234	69.136	62.328	83.566	63.005	19.691
676	99.799	69.224	62.364	83.159	63.42	19.87
677	100.284	69.458	62.739	83.303	63.529	19.868
678	100.126	69.602	62.686	83.457	63.699	20.196
679	100.278	69.712	62.915	83.165	64.128	20.258
680	100.395	69.911	63.049	83.584	64.265	20.514
681	99.827	69.914	62.956	83.077	64.375	20.434
682	99.846	69.897	63.006	83.324	64.288	20.615
683	99.86	70.187	63.277	83.406	64.874	20.818
684	100.436	70.431	63.321	83.54	65.183	20.95
685	100.215	70.272	63.769	83.549	65.17	20.97
686	100.19	70.851	63.976	83.707	65.706	21.351
687	100.434	70.932	64.152	83.623	65.779	21.319
688	100.007	70.422	64.164	83.528	65.867	21.298
689	100.295	71.073	64.517	83.628	66.333	21.522
690	99.958	70.899	64.34	83.285	66.127	21.744
691	100.219	71.301	64.256	83.381	66.373	21.819
692	99.825	71.335	64.634	83.465	66.763	21.824
693	100.05	71.507	65.171	83.632	67.194	21.966
694	100.357	71.617	64.962	83.886	67.4	22.268
695	100.318	71.486	65.14	83.832	67.313	22.466
696	100.047	71.858	65.066	83.439	67.984	22.432
697	99.986	71.937	65.223	83.307	67.801	22.525
698	99.716	71.775	65.552	83.809	68.062	22.644
699	100.225	72.017	65.678	83.733	68.074	22.99
		**Anowar_SI_NPND_5_RMIT**				

Open Researcher and Contributor ID (ORCID) Md. Anowar Hossain: https://orcid.org/0000-0003-2880-6287

Table 6. Supporting information of reflection (%) from 700 nm to 799 nm for standardized barium sulphate, *Swietenia Macrophylla* dyed fabric without mordanting, *Swietenia Macrophylla* dyed fabric with mordanting, undyed knitted fabric, raw *Swietenia Macrophylla* and raw *Areca Catechu*.

Wavelength (nm)	Standardized Barium Sulphate	Swietenia Macrophylla dyed fabric without mordanting	Swietenia Macrophylla dyed fabric with mordanting	Undyed knitted fabric	Swietenia Macrophylla	Raw Areca Catechu
700	100.114	72.189	65.737	83.86	68.599	23.139
701	100.369	72.678	66.032	84.005	68.779	23.302
702	100.317	72.311	66.078	84.107	68.899	23.235
703	100.767	72.634	66.275	84.217	68.841	23.441
704	100.225	72.67	66.374	83.67	69.24	23.716
705	100.323	72.876	66.218	84.042	69.09	23.809
706	100.181	72.909	66.409	84.187	69.511	23.722
707	100.581	72.854	66.594	83.873	69.511	24.042
708	100.487	73.087	66.94	84.217	69.807	24.124
709	100.391	73.342	66.761	84.048	69.909	24.171
710	101.138	73.633	67.267	84.183	70.239	24.382
711	100.432	73.405	67.311	84.039	70.462	24.467
712	100.375	73.757	67.389	83.788	70.481	24.695
713	100.482	73.557	67.407	84.014	70.449	24.887
714	100.524	73.578	67.568	84.187	70.793	24.976
715	100.764	73.733	67.809	84.036	71.071	25.093
716	99.793	73.835	67.543	84.359	71.244	25.068
717	100.066	74.243	68.148	83.855	71.406	25.234
718	99.857	74.074	67.986	84.215	71.734	25.356
719	99.905	74.079	67.987	84.595	71.591	25.56
720	100.038	74.468	68.097	84.265	71.692	25.666
721	100.154	74.385	67.983	84.348	72.017	25.693
722	100.23	74.872	68.443	84.448	71.981	25.993
723	100.163	74.374	68.694	84.195	72.071	26.104
724	100.337	74.703	68.759	84.687	72.221	26.141
725	99.811	74.578	68.58	84.34	72.397	26.302
726	100.041	74.898	68.71	84.357	72.351	26.475
727	99.899	74.808	68.846	84.136	72.623	26.535
728	99.958	74.891	68.654	84.152	72.494	26.736
729	100.24	74.78	69.551	84.886	72.938	26.774
730	99.902	74.977	69.43	84.526	73.114	27.004
731	100.191	75.8	69.349	84.965	73.307	27.092
732	99.668	75.451	69.041	84.623	72.982	27.358
733	100.481	75.486	69.512	84.437	73.636	27.471
734	100.121	75.522	69.656	84.334	73.406	27.391
735	99.907	75.921	70.008	84.436	73.321	27.515
736	100.326	75.526	69.841	84.615	74.064	27.728
737	100.206	75.776	70.432	84.847	74.263	27.737
738	99.828	75.889	69.694	84.167	74.033	27.611
739	100.132	76.282	70.489	84.684	74.576	28.027
740	100.364	76.2	70.29	84.6	74.355	28.396
741	100.452	76.274	70.202	84.941	74.628	28.495
742	100.165	76.357	70.305	84.899	74.579	28.241
743	100.255	76.346	70.987	85.359	74.992	28.67
744	100.291	76.33	70.938	85.229	75.214	28.514
745	99.978	76.708	70.849	85.232	75.161	28.823
746	99.658	76.845	71.42	84.81	75.199	28.912
747	99.859	76.254	71.115	85.082	75.195	29.186
748	99.873	76.374	70.753	85.621	75.617	29.013
749	100.197	76.591	71.11	84.929	75.676	29.459
750	100.062	76.146	71.116	84.912	75.524	29.237
751	100.064	76.478	71.173	85.282	75.4	29.512
752	99.878	77.009	71.164	84.655	75.959	29.396
753	100.233	77.657	71.231	85.664	76.239	29.561
754	100.167	77.286	72.256	85.643	76.584	29.945
755	100.426	77.453	71.643	85.969	76.157	30.087
756	99.9	77.351	71.587	85.328	76.654	30.169
757	99.894	77.41	72.081	85.318	76.256	30.211
758	100.171	77.709	72.135	85.834	76.761	30.459
759	100.559	78.205	72.477	85.784	77.223	30.397
760	99.415	77.448	72.206	85.154	76.021	30.579
761	99.736	78.036	72.412	85.384	77.242	30.818
762	99.268	77.505	72.416	84.639	76.795	30.809
763	99.528	77.777	72.181	85.502	76.925	30.698
764	99.751	78.432	72.735	85.351	77.564	31.089
765	100.731	78.313	73.128	86.35	77.879	31.71
766	100.008	78.329	73.158	86.019	77.343	31.465
767	99.34	78.04	71.946	85.167	76.833	31.649
768	100.232	78.788	72.738	85.974	77.433	32.023
769	99.845	78.07	72.695	85.381	76.852	31.847
770	99.606	78.555	73.31	85.713	77.465	31.673
771	99.859	77.728	72.897	84.979	77.178	31.707
772	100.659	78.299	73.424	86.082	78.021	32.242
773	98.798	78.103	72.597	85.11	77.199	32.227
774	99.749	78.173	73.075	85.311	77.254	32.597
775	99.207	78.794	73.269	85.08	77.895	32.377
776	100.225	79.392	74.158	86.447	78.621	32.974
777	99.816	79.996	74.3	86.73	78.105	33.042
778	99.844	78.902	73.326	85.815	78.488	32.879
779	99.4	78.531	73.787	85.355	78.156	33.09
780	100.299	79.647	74.446	86.207	78.678	32.912
781	100.489	79.328	74.197	85.964	78.608	33.265
782	100.121	79.038	74.844	86.259	78.711	33.539
783	99.645	79.329	74.34	86.373	78.515	33.773
784	99.591	80.02	74.212	86.144	78.629	33.683
785	100.139	79.283	75.16	85.702	79.262	33.966
786	99.935	79.428	74.423	85.609	78.832	34.103
787	100.05	79.99	74.255	85.557	79.494	34.17
788	98.952	79.727	74.304	85.117	79.026	33.821
789	99.752	78.718	74.845	85.303	78.661	34.102
790	100.42	79.587	74.833	85.88	79.048	34.346
791	99.351	79.573	74.525	86.062	78.833	34.621
792	100.136	79.669	74.81	86.158	79.515	35.026
793	99.341	80.348	75.373	86.155	79.665	35.094
794	100.916	79.387	75.43	86.108	79.146	34.666
795	100.455	79.964	75.289	86.514	79.693	35.037
796	101.316	80.291	75.542	86.665	80.362	35.768
797	99.611	80.649	75.147	86.347	79.795	35.357
798	99.54	79.656	75.696	86.101	79.899	35.163
799	100.098	80.336	75.556	86.275	80.159	35.76
		**Anowar_SI_NPND_6_RMIT**				

Open Researcher and Contributor ID (ORCID) Md. Anowar Hossain: https://orcid.org/0000-0003-2880-6287

Table 7. Supporting information of reflection (%) from 800 nm to 899 nm for standardized barium sulphate, *Swietenia Macrophylla* dyed fabric without mordanting, *Swietenia Macrophylla* dyed fabric with mordanting, undyed knitted fabric, raw *Swietenia Macrophylla* and raw *Areca Catechu*.

Wavelength (nm)	Standardized Barium Sulphate	Swietenia Macrophylla dyed fabric without mordanting	Swietenia Macrophylla dyed fabric with mordanting	Undyed knitted fabric	Swietenia Macrophylla	Raw Areca Catechu
800	100.619	80.026	75.73	86.162	79.828	35.554
801	99.899	80.994	75.944	85.839	80.005	36
802	100.07	80.74	76.139	87.339	80.104	35.684
803	100.988	81.171	76.412	86.871	81.133	36.438
804	100.126	81.396	76.105	85.759	80.704	36.379
805	100.658	81	76.579	87.117	81.034	36.554
806	100.845	80.618	76.372	86.872	80.227	36.173
807	101.066	81.566	76.686	87.217	81.137	36.678
808	100.795	81.968	77.578	86.673	81.247	36.821
809	100.191	80.945	76.811	85.979	80.264	36.876
810	100.566	80.926	76.45	86.023	80.202	36.754
811	100.219	81.001	77.447	86.645	80.877	37.012
812	100.031	82.5	76.541	86.466	80.857	37.416
813	100.011	81.979	77.148	86.521	80.845	37.081
814	100.82	81.564	76.633	87.033	81.498	37.591
815	100.148	81.193	76.736	86.65	80.954	37.683
816	101.022	83.156	78.165	86.72	82.502	38.149
817	100.353	81.665	76.926	86.943	81.628	38.158
818	100.278	81.738	77.325	86.128	81.334	37.643
819	100.263	81.474	77.214	86.065	81.227	37.835
820	100.555	81.74	77.033	86.417	81.337	37.759
821	99.572	81.868	77.347	86.935	82.112	37.905
822	100.554	81.199	77.921	87.191	81.695	38.65
823	101.882	83.2	78.454	88.088	82.815	39.482
824	99.412	80.686	78.1	86.265	81.282	38.326
825	100.579	82.575	78.234	86.967	82.209	38.884
826	100.763	82.934	78.435	86.447	82.547	39.216
827	99.793	82.596	77.578	86.562	81.94	38.859
828	99.616	81.715	77.271	86.054	81.44	39.115
829	100.136	81.475	77.632	86.702	82.013	38.984
830	100.608	82.794	78.349	86.985	82.588	40.01
831	100.565	82.832	79.497	88.671	83.206	40.893
832	101.807	82.438	79.311	86.524	82.907	40.858
833	99.974	83.473	78.972	87.095	82.7	40.464
834	101.165	83.57	79.559	87.367	84.368	42.04
835	99.696	82.28	80.196	87.353	82.079	42.221
836	100.407	82.206	77.292	87.081	82.002	40.201
837	100.563	83.265	78.735	87.704	82.851	41.087
838	100.626	83.158	79.159	87.685	82.041	40.981
839	99.051	82.756	80.01	85.978	82.375	41.603
840	101.497	83.225	80.976	87.485	82.863	41.647
841	99.372	82.335	79.446	87.356	82.468	40.513
842	99.69	82.093	79.531	87.089	83.562	41.24
843	99.049	82.873	80.611	87.552	82.454	41.529
844	99.976	82.14	80.147	87.413	82.99	42.273
845	99.443	81.011	79.897	86.802	82.559	41.755
846	100.03	81.576	79.354	85.14	82.54	41.364
847	100.195	82.448	78.997	86.01	82.579	41.876
848	98.845	82.112	80.137	86.643	83.591	42.427
849	99.765	84.176	80.466	86.83	84.225	42.597
850	98.758	82.633	79.32	86.599	82.163	42.062
851	100.327	82.967	79.646	86.629	83.801	42.442
852	101.54	84.358	80.558	87	84.626	43.037
853	100.164	83.144	80.984	86.4	82.849	42.468
854	99.417	81.891	79.274	85.55	82.784	41.99
855	99.946	83.149	80.292	88.52	83.87	43.404
856	100.499	82.795	80.493	86.114	83.393	44.062
857	101.537	82.689	81.166	86.926	83.096	43.473
858	100.24	83.923	81.122	86.629	84.167	43.93
859	101.75	82.956	79.814	87.207	84.306	44.089
860	100.789	83.418	81.041	87.705	82.847	44.079
861	100.122	83.848	81.441	87.867	83.203	44.573
862	100.342	83.932	80.754	86.692	83.873	43.357
863	100.748	82.896	81.301	86.708	83.132	43.873
864	100.04	84.263	81.1	87.882	84.304	44.454
865	100.766	83.899	82.209	87.374	84.604	44.548
866	99.796	82.895	80.708	87	82.717	43.647
867	100.086	84.351	80.623	86.912	84.386	43.855
868	100.418	84.445	81.326	86.368	83.877	44.521
869	99.451	83.856	81.136	86.953	83.894	44.584
870	101.317	84.23	81.738	87.432	83.804	44.734
871	101.035	85.256	82.204	87.671	84.929	45.119
872	99.93	85.641	83.168	87.962	83.848	45.231
873	100.536	84.832	81.748	87.146	84.239	45.386
874	101.155	85.059	83.037	87.731	85.173	44.86
875	101.17	84.882	82.394	88.436	84.163	45.239
876	99.731	82.151	80.713	86.606	83.659	45.352
877	100.579	84.919	81.754	86.863	85.169	45.271
878	100.868	85.429	82.767	88.47	85.592	46.635
879	100.026	84.149	82.145	86.357	83.941	46.007
880	100.42	84.968	82.96	86.992	85.043	46.82
881	101.324	84.969	82.8	87.798	85.701	46.306
882	100.781	85.729	82.639	88.183	85.293	46.644
883	99.633	84.677	82.065	86.779	85.16	46.487
884	99.52	84.017	81.88	86.863	84.436	46.264
885	100.433	84.302	82.17	87.35	84.59	45.945
886	101.095	84.775	82.891	87.518	85.164	47.145
887	100.628	84.921	82.765	87.651	84.985	46.833
888	99.858	84.063	82.273	86.899	84.064	47.105
889	100.35	84.906	82.28	88.666	84.335	46.424
890	100.581	85.124	83.601	87.569	85.074	46.945
891	100.112	84.337	82.513	88.036	83.964	46.537
892	100.388	84.235	82.123	86.517	84.268	47.285
893	100.538	85.096	82.379	87.173	84.279	47.211
894	100.367	84.801	82.476	87.607	85.676	47.35
895	99.916	85.356	83.031	87.449	85.301	46.995
896	99.808	85.382	82.842	86.979	84.568	47.272
897	99.961	84.499	82.718	86.53	85.263	47.57
898	99.545	85.1	82.773	87.18	85.53	47.893
899	100.41	85.595	82.889	87.327	85.241	47.226
		**Anowar_SI_NPND_7_RMIT**				

Open Researcher and Contributor ID (ORCID) Md. Anowar Hossain: https://orcid.org/0000-0003-2880-6287

Table 8. Supporting information of reflection (%) from 900 nm to 999 nm for standardized barium sulphate, *Swietenia Macrophylla* dyed fabric without mordanting, *Swietenia Macrophylla* dyed fabric with mordanting, undyed knitted fabric, raw *Swietenia Macrophylla* and raw *Areca Catechu*.

Wavelength (nm)	Standardized Barium Sulphate	Swietenia Macrophylla dyed fabric without mordanting	Swietenia Macrophylla dyed fabric with mordanting	Undyed knitted fabric	Swietenia Macrophylla	Raw Areca Catechu
900	99.81	84.987	82.625	87.02	85.28	48.042
901	102.101	86.094	83.436	88.459	85.883	47.784
902	99.455	85.305	82.797	87.198	84.542	47.924
903	99.705	83.898	82.661	86.982	84.282	48.138
904	99.377	84.921	83.002	87.078	85.208	48.029
905	101.181	85.311	83.503	88.17	85.663	48.672
906	99.892	84.506	83.157	87.121	84.482	48.236
907	99.877	85.751	83.138	87.585	84.924	48.023
908	100.045	85.191	82.569	87.367	84.861	48.183
909	99.921	85.198	82.29	87.423	84.83	48.282
910	100.829	85.828	83.837	88.01	85.078	49.109
911	100.388	85.305	83.104	88.032	85.147	49.649
912	99.622	84.396	83.302	87.355	85.031	48.784
913	100.214	84.93	83.016	87.276	84.628	48.79
914	100.162	85.879	83.705	87.939	85.339	48.907
915	101.144	85.483	83.02	87.535	85.588	48.976
916	100.464	85.649	83.88	87.576	86.076	49.372
917	100.483	85.363	83.628	87.69	85.378	49.667
918	100.362	85.785	83.535	87.637	85.351	49.57
919	100.473	85.853	83.826	87.693	85.019	49.894
920	100.789	85.412	83.342	87.216	85.956	49.414
921	100.096	85.448	83.839	87.108	85.073	50.001
922	100.058	85.408	83.596	87.328	84.571	49.544
923	99.864	84.872	84.087	87.453	85.252	49.697
924	100.293	85.512	84.125	87.632	85.363	49.909
925	99.677	85.443	84.182	87.731	85.608	50.096
926	100.74	86.342	84.184	87.708	86.066	50.372
927	100.593	86.09	84.166	88.333	85.637	50.663
928	100.337	85.248	83.958	87.208	85.488	50.695
929	100.152	85.851	84.777	88.206	85.798	50.346
930	100.703	85.451	84.301	88.008	85.872	50.528
931	100.469	85.709	83.931	88.127	85.58	50.609
932	100.623	85.889	84.876	87.975	86.655	51.386
933	100.039	86.513	84.347	87.95	85.768	50.385
934	100.434	85.895	84.459	87.641	85.682	50.793
935	100.155	85.99	84.392	87.766	85.504	51.064
936	100.419	85.955	84.4	88.007	85.478	51.139
937	100.352	86.166	84.732	88.047	85.696	51.595
938	99.662	86.186	84.414	87.178	85.531	51.2
939	100.421	86.138	84.789	88.538	86.097	51.677
940	100.971	85.804	84.734	87.808	85.682	51.72
941	99.734	85.612	84.728	87.505	85.526	51.739
942	100.241	86.202	84.328	87.592	85.58	51.361
943	99.773	86.252	84.182	87.441	85.868	51.48
944	100.088	86.573	85.053	88.04	86.167	51.825
945	100.59	86.267	84.553	88.262	85.466	51.774
946	99.988	86.243	84.881	88.184	86.09	51.716
947	100.05	86.199	84.891	87.902	85.962	51.758
948	100.251	85.826	85.066	87.798	86.007	52.316
949	99.868	85.688	84.771	87.579	85.607	51.624
950	101.087	86.977	85.24	89.083	86.896	52.641
951	99.86	86.373	85.352	87.953	86.183	52.331
952	100.951	87.056	85.228	88.501	86.082	52.799
953	100.203	86.206	85.456	88.116	86.343	52.456
954	100.296	86.834	85.372	88.609	86.006	52.685
955	99.971	86.351	85.059	88.508	86.445	52.852
956	99.82	86.537	85.616	87.631	85.887	52.801
957	100.219	86.908	85.425	88.062	85.854	53.253
958	99.954	86.379	84.898	87.882	86.065	53.127
959	99.908	86.648	84.789	87.662	85.503	52.984
960	100.474	86.525	84.91	88.052	85.702	53.058
961	100.192	86.65	85.92	88.317	86.313	53.534
962	99.916	86.249	85.263	87.353	85.871	52.65
963	100.319	86.643	84.898	87.777	86.307	53.664
964	100.215	86.537	84.929	87.784	85.557	53.099
965	100.009	86.499	85.473	88.351	86.31	54.003
966	100.681	86.651	85.952	88.767	86.157	53.441
967	100.253	86.391	85.488	87.802	86.632	53.588
968	100.075	87.143	85.594	88.192	86.198	53.959
969	99.798	86.611	85.538	88.145	86.188	53.971
970	100.334	86.526	85.712	87.918	85.607	53.445
971	100.32	86.505	85.532	87.892	86.109	53.821
972	100.394	86.98	85.315	88.109	86.014	54.136
973	100.268	86.635	85.636	88.257	86.25	54.258
974	100.429	86.781	85.457	88.312	85.974	54.005
975	99.634	86.536	85.204	88.137	85.848	53.827
976	99.783	86.246	85.94	88.041	85.678	54.267
977	100.876	87.129	86.28	88.457	86.221	54.704
978	100.596	86.701	85.74	88.337	86.347	54.369
979	100.313	86.422	85.866	88.472	85.848	54.896
980	99.86	86.23	85.853	88.247	85.55	55.059
981	100.935	87.259	86.208	88.195	86.114	55.166
982	100.617	86.192	85.651	88.206	85.659	54.244
983	99.887	86.362	85.192	87.722	85.82	54.6
984	99.561	86.076	85.513	87.515	85.476	54.746
985	100.317	86.358	86.2	88.184	86.074	55.324
986	100.442	86.813	86.2	88.177	86.622	55.354
987	99.8	86.725	85.602	88.296	86.241	55.257
988	99.83	86.948	85.837	87.769	85.734	55.225
989	99.919	86.736	85.714	88.209	86.083	55.351
990	100.009	86.213	86.024	87.483	85.921	55.523
991	100.382	86.963	86.087	88.236	86.511	55.691
992	99.666	86.637	85.886	87.846	86.147	55.6
993	100.63	87.022	86.21	88.12	86.186	55.46
994	99.859	86.4	85.496	87.46	85.575	55.002
995	100.351	86.929	85.962	88.047	86.204	56.201
996	99.873	86.642	85.755	87.336	85.989	55.702
997	100.256	86.451	85.729	87.907	85.853	55.861
998	100.169	86.843	85.946	87.896	86.124	56.294
999	99.862	86.645	86.188	87.942	85.724	56.188
		**Anowar_SI_NPND_8_RMIT**				

Open Researcher and Contributor ID (ORCID) Md. Anowar Hossain: https://orcid.org/0000-0003-2880-6287

Table 9. Supporting information of reflection (%) from 1000 nm to 1099 nm for standardized barium sulphate, *Swietenia Macrophylla* dyed fabric without mordanting, *Swietenia Macrophylla* dyed fabric with mordanting, undyed knitted fabric, raw *Swietenia Macrophylla* and raw *Areca Catechu*.

Wavelength (nm)	Standardized Barium Sulphate	Swietenia Macrophylla dyed fabric without mordanting	Swietenia Macrophylla dyed fabric with mordanting	Undyed knitted fabric	Swietenia Macrophylla	Raw Areca Catechu
1000	100.239	87.256	86.336	88.649	86.354	56.432
1001	99.673	86.699	86.23	88.094	86.148	56.572
1002	99.67	86.981	85.983	87.913	86.187	56.737
1003	99.878	86.48	86.169	88.043	85.773	56.568
1004	99.689	86.331	85.311	87.682	85.597	56.686
1005	99.802	86.735	86.376	88.236	86.637	56.452
1006	100.305	86.7	86.706	88.061	86.634	56.919
1007	100.106	86.26	86.271	87.834	86.196	56.575
1008	99.78	86.709	85.822	87.946	86.173	56.266
1009	100.146	86.617	86.117	88.239	86.054	56.873
1010	100.118	86.464	86.227	88.041	86.245	57.269
1011	100.345	86.679	86.226	87.515	86.148	56.593
1012	99.826	86.374	86.478	87.893	86.348	57.276
1013	99.854	86.452	86.192	87.49	86.326	56.814
1014	100.411	87.054	86.813	88.042	86.677	57.342
1015	99.865	87.072	86.754	87.999	86.391	57.659
1016	100.485	87.016	86.442	88.106	86.376	57.457
1017	100.606	87.212	86.807	88.487	86.751	57.756
1018	100.107	86.985	86.571	88.234	86.466	57.639
1019	100.043	86.908	87.041	87.995	86.311	57.872
1020	100.226	86.596	86.445	88.004	87.072	57.886
1021	99.751	86.758	86.631	88.302	86.472	57.485
1022	99.995	86.923	86.825	87.614	86.485	57.749
1023	100.016	86.838	86.114	87.974	86.759	58.207
1024	100.153	87.122	86.557	88.104	86.591	58.063
1025	100.041	86.9	86.513	88.213	86.733	58.299
1026	99.911	86.878	86.732	88.302	86.714	58.802
1027	100.906	87.664	87.144	88.265	87.041	58.536
1028	99.722	86.642	86.788	87.88	86.324	58.666
1029	100.036	86.953	86.847	88.325	86.719	58.328
1030	100.436	87.244	86.562	88.666	86.712	58.957
1031	100.218	87.141	86.868	88.311	86.674	58.823
1032	99.882	87.135	86.671	88.155	86.509	58.584
1033	100.273	87.625	86.939	88.619	87.111	59.203
1034	100.424	87.062	87.225	88.506	86.894	59.502
1035	100.347	87.355	86.871	88.536	86.94	59.377
1036	100.251	86.999	86.803	88.216	86.192	59.019
1037	100.271	87.769	86.9	88.677	87.485	59.245
1038	100.021	86.868	87.168	88.091	86.487	59.181
1039	100.367	87.095	87.045	88.159	86.751	59.355
1040	100.193	87.097	87.256	88.384	86.94	59.225
1041	100.002	87.26	86.98	88.487	86.985	59.181
1042	99.945	87.546	86.777	88	86.429	59.495
1043	100.437	87.717	87.357	88.983	87.218	59.981
1044	100.041	87.383	87.121	88.454	87.74	59.386
1045	100.365	87.208	87.431	88.289	86.869	59.374
1046	100.141	87.061	87.081	88.454	86.478	59.917
1047	100.372	87.123	87.221	88.396	87.064	59.866
1048	100.52	87.208	87.02	88.244	87.02	60.243
1049	100.204	87.485	87.101	88.07	86.784	59.944
1050	100.186	87.472	87.251	88.216	87.216	60.263
1051	100.482	87.437	87.231	88.299	87.375	60.185
1052	100.546	87.651	87.5	88.757	87.371	60.512
1053	100.024	87.674	87.691	88.613	87.365	60.436
1054	99.989	87.567	87.753	88.491	87.188	60.504
1055	100.241	87.63	87.566	88.251	86.839	60.646
1056	100.388	87.556	87.59	88.64	87.115	60.384
1057	99.993	87.784	87.885	88.588	87.372	60.753
1058	100.494	87.798	87.781	88.701	87.314	60.811
1059	100.046	87.692	87.246	88.497	87.317	60.474
1060	100.201	87.521	87.824	88.784	87.237	61.168
1061	100.28	87.805	87.934	88.924	87.511	61.128
1062	99.696	87.181	87.377	88.51	87.043	61.252
1063	100.074	87.728	88.239	89.038	87.274	61.581
1064	100.509	87.831	88.186	89.15	87.356	61.812
1065	100.093	88.005	87.954	88.727	87.549	61.422
1066	99.869	87.416	87.387	88.771	87.164	61.232
1067	99.846	87.688	87.318	88.824	87.146	61.395
1068	99.743	87.641	87.944	88.591	87.059	61.397
1069	99.869	87.715	87.783	88.682	87.375	61.285
1070	100.033	88.079	87.764	88.847	87.397	61.707
1071	99.853	87.851	88.183	88.683	86.937	61.759
1072	100.257	88.402	88.258	89.066	87.571	61.67
1073	99.695	87.952	87.906	88.347	87.438	61.919
1074	100.447	88.201	88.832	89.203	87.758	62.317
1075	100.182	87.727	87.989	89.076	87.159	62.119
1076	99.952	87.875	88.689	88.874	87.725	62.842
1077	100.252	88.11	87.968	88.708	87.233	62.571
1078	100.557	88.118	87.88	89.215	87.67	62.544
1079	100.091	87.632	88.09	88.575	87.235	62.568
1080	100.545	87.954	88.579	89.409	87.692	62.829
1081	100.288	87.982	88.139	89.023	87.469	62.403
1082	99.888	87.948	88.255	89.004	87.648	62.663
1083	99.727	87.932	87.726	88.904	87.345	62.753
1084	99.469	87.574	87.599	88.405	86.93	62.498
1085	100.153	87.966	88.512	89.022	87.817	62.907
1086	100.39	87.947	88.4	89.043	87.398	62.944
1087	100.549	88.237	88.7	89.135	87.915	63.358
1088	99.881	87.663	88.169	88.757	87.368	63.15
1089	100.527	88.402	88.131	88.75	87.761	63.27
1090	100.42	88.134	88.59	89.442	87.661	63.589
1091	100.029	88.07	88.397	88.713	87.432	63.586
1092	100.014	88.264	88.683	88.96	87.833	63.516
1093	99.847	88.036	88.52	89.357	87.733	63.551
1094	99.939	88.131	88.081	88.994	87.295	63.452
1095	99.997	88.229	88.662	88.926	87.799	63.554
1096	100.532	88.861	88.734	89.518	87.829	63.625
1097	100.605	88.658	88.929	88.766	87.676	63.763
1098	99.735	88.334	88.429	89.072	87.413	63.9
1099	99.76	88.291	89.049	88.933	87.61	63.879
		Anowar_SI_NPND_9_RMIT				

Open Researcher and Contributor ID (ORCID) Md. Anowar Hossain: https://orcid.org/0000-0003-2880-6287

Table 10. Supporting information of reflection (%) from 1100 nm to 1199 nm for standardized barium sulphate, *Swietenia Macrophylla* dyed fabric without mordanting, *Swietenia Macrophylla* dyed fabric with mordanting, undyed knitted fabric, raw *Swietenia Macrophylla* and raw *Areca Catechu*.

Wavelength (nm)	Standardized Barium Sulphate	Swietenia Macrophylla dyed fabric without mordanting	Swietenia Macrophylla dyed fabric with mordanting	Undyed knitted fabric	Swietenia Macrophylla	Raw Areca Catechu
1100	99.618	88.043	88.476	88.668	87.545	64.014
1101	100.129	88.614	89.094	89.569	87.72	64.739
1102	99.984	88.175	88.987	89.346	87.731	64.334
1103	100.34	88.665	89.235	89.209	87.552	64.538
1104	99.886	88.74	89.003	89.27	87.677	64.652
1105	99.752	88.399	89.288	89.235	87.731	64.19
1106	99.545	88.5	88.881	89.32	87.352	63.863
1107	99.844	88.291	88.637	89.17	87.573	64.751
1108	100.314	88.973	89.192	89.814	88.448	64.779
1109	99.8	88.544	88.86	89.002	87.54	64.37
1110	100.336	88.751	89.394	89.85	88.047	64.9
1111	99.905	88.522	89.595	89.516	88.144	64.906
1112	100.471	88.456	89.356	89.803	87.848	65.291
1113	100.252	88.841	89.137	89.577	87.436	65.211
1114	100.279	88.551	89.308	89.42	88.131	65.414
1115	99.987	88.491	88.766	89.269	87.431	64.934
1116	100.235	89.006	89.381	89.496	87.99	65.172
1117	99.558	88.444	89.073	89.313	87.589	65.359
1118	100.28	88.431	89.249	89.636	87.881	65.242
1119	100.145	88.542	89.209	89.288	87.29	65.375
1120	100.576	88.669	89.491	89.676	88.591	65.771
1121	100.052	89.125	89.558	89.511	87.532	66.115
1122	99.719	88.735	88.779	89.078	87.658	65.856
1123	100.346	89.366	89.406	90.012	88.142	66.005
1124	100.666	89.534	89.807	90.137	87.778	66.144
1125	100.146	89.202	89.331	89.557	87.17	65.48
1126	99.995	88.919	89.133	89.754	87.063	66.291
1127	99.945	88.791	89.47	89.466	87.264	66.171
1128	100.651	89.346	89.842	90.043	87.828	66.864
1129	99.952	89.039	89.01	89.857	87.83	66.398
1130	99.882	88.684	88.75	89.615	87.35	65.925
1131	99.982	88.708	89.069	89.835	87.547	66.592
1132	100.019	89.299	89.804	89.519	87.663	66.434
1133	100.33	89.248	89.688	89.918	87.303	66.833
1134	99.799	89.248	89.549	90.068	87.951	66.668
1135	100.345	89.464	89.788	89.824	87.494	66.281
1136	99.863	88.665	89.429	89.811	87.288	66.998
1137	100.383	88.898	89.703	89.789	87.613	67.283
1138	99.954	88.682	89.704	89.693	87.276	66.93
1139	100.404	89.282	89.763	89.797	87.372	66.619
1140	100.268	88.838	89.662	89.578	87.501	67.376
1141	99.987	89.409	89.578	89.634	87.331	67.019
1142	99.933	88.428	89.177	89.373	87.022	66.991
1143	99.572	88.337	89.458	89.439	86.906	66.994
1144	100.005	88.847	89.375	89.269	86.918	67.136
1145	100.201	88.821	89.225	89.193	86.721	67.45
1146	99.952	88.234	89.169	89.267	86.815	67.26
1147	100.131	88.787	89.269	89.104	86.956	67.502
1148	100.642	88.736	89.761	89.849	87.28	67.704
1149	99.902	88.878	89.187	89.179	86.805	67.666
1150	100.195	88.271	89.487	89.059	86.839	67.618
1151	99.932	88.509	89.113	88.852	86.45	67.815
1152	100.067	88.382	89.03	89.259	86.809	68.065
1153	99.701	88.481	88.994	89.014	86.478	67.436
1154	100.309	87.821	89.199	89.163	86.352	68.068
1155	100.008	87.972	89.186	89.167	86.882	68.102
1156	99.77	87.995	88.752	89.006	86.715	68.122
1157	99.897	88.151	88.826	88.685	86.386	67.864
1158	99.899	87.914	88.779	88.815	86.509	67.923
1159	99.913	88.478	89.264	89.077	86.746	68.846
1160	100.201	88.607	89.518	88.91	87.126	68.322
1161	100.196	87.72	88.36	88.585	86.198	68.027
1162	100.577	87.872	89.121	88.612	87.357	68.882
1163	99.379	87.832	88.427	87.777	86.452	68.628
1164	99.96	87.566	88.543	88.258	85.938	68.576
1165	100.251	87.623	88.346	88.437	86.636	68.649
1166	100.139	87.596	88.395	88.32	86.413	68.642
1167	100.262	88.047	89.004	88.747	86.681	69.068
1168	100.336	87.89	88.857	88.11	86.26	68.74
1169	100.346	87.948	88.943	88.51	86.229	68.801
1170	100.281	87.31	88.358	88.272	86.206	69.105
1171	99.661	87.14	87.816	87.563	85.997	68.944
1172	100.117	87.512	88.733	88.223	85.91	68.938
1173	99.708	87.801	88.85	88.284	86.427	69.232
1174	100.205	86.764	88.168	87.758	85.84	69.219
1175	99.572	87.048	87.753	87.585	85.528	68.911
1176	100.525	87.077	88.647	88.059	86.36	69.433
1177	100.75	87.144	88.326	87.82	86.227	69.841
1178	99.837	86.775	88.064	87.663	85.846	69.684
1179	100.47	86.606	88.204	87.601	86.558	69.785
1180	100.115	86.261	87.606	87.18	85.854	69.532
1181	99.955	86.383	87.584	87.329	86.003	69.462
1182	100.385	86.616	87.401	86.898	85.738	70.079
1183	99.971	86.325	87.549	86.759	85.854	69.558
1184	100.08	86.147	87.589	86.994	85.559	70.177
1185	99.963	85.809	87.096	86.772	85.864	69.754
1186	100.374	86.082	87.291	86.667	85.626	69.885
1187	100.045	85.662	87.399	86.771	85.589	70.293
1188	99.831	85.485	87.189	86.416	85.79	70.111
1189	100.079	86.022	87.545	87.055	86.1	71.097
1190	99.968	85.869	86.664	86.76	85.562	70.528
1191	100.056	85.808	86.833	86.331	85.937	70.497
1192	100.224	85.131	86.386	86.263	86.015	70.183
1193	99.696	85.333	86.36	85.977	85.207	70.755
1194	100.137	85.331	86.658	86.382	85.737	71.087
1195	100.539	85.124	86.568	86.158	85.819	70.911
1196	100.219	85.76	86.672	86.401	85.831	71.322
1197	100.027	85.086	86.588	85.616	85.713	70.943
1198	99.815	85.498	86.515	86.13	85.581	71.107
1199	100.146	85.49	86.663	85.814	85.735	71.058
		**Anowar_SI_NPND_10_RMIT**				

Open Researcher and Contributor ID (ORCID) Md. Anowar Hossain: https://orcid.org/0000-0003-2880-6287

Table 11. Supporting information of reflection (%) from 1200 nm to 1299 nm for standardized barium sulphate, *Swietenia Macrophylla* dyed fabric without mordanting, *Swietenia Macrophylla* dyed fabric with mordanting, undyed knitted fabric, raw *Swietenia Macrophylla* and raw *Areca Catechu*.

Wavelength (nm)	Standardized Barium Sulphate	Swietenia Macrophylla dyed fabric without mordanting	Swietenia Macrophylla dyed fabric with mordanting	Undyed knitted fabric	Swietenia Macrophylla	Raw Areca Catechu
1200	99.569	84.718	86.402	85.658	85.51	71.163
1201	100.661	84.83	86.401	85.681	85.899	71.347
1202	99.897	84.993	86.349	85.596	85.865	71.109
1203	100.075	85.259	86.521	85.736	86.118	71.388
1204	99.711	85.423	86.215	85.585	86.087	71.446
1205	99.787	84.388	86.112	85.08	85.528	71.303
1206	99.828	84.449	85.885	85.038	85.385	71.323
1207	100.194	84.713	86.286	85.147	85.521	71.803
1208	99.885	84.862	86.24	85.145	85.924	71.419
1209	100.423	84.736	86.246	85.442	85.917	71.84
1210	100.188	84.7	85.787	85.194	86.254	72.25
1211	100.204	84.544	85.912	85.477	85.735	71.682
1212	100.036	84.349	86.396	85.254	86.031	72.204
1213	100.451	85.137	86.276	85.817	86.084	72.641
1214	99.452	84.096	85.878	83.821	85.214	72.033
1215	99.662	83.869	85.237	84.796	85.07	72.269
1216	100.24	84.629	86.057	85.372	86.209	73.089
1217	100.152	84.501	86.225	84.895	86.579	72.736
1218	100.101	84.21	85.799	84.916	86.279	72.869
1219	100.586	84.81	85.869	84.798	86.026	72.755
1220	99.718	83.998	86.011	85.005	86.061	72.843
1221	100.426	84.669	86.097	85.02	86.517	72.847
1222	100.414	84.64	86.042	85.009	86.055	73.232
1223	99.88	84.928	86.024	84.616	85.997	73.61
1224	99.715	84.543	85.663	84.727	86.263	73.222
1225	100.18	84.913	85.875	85.022	86.232	73.409
1226	100.091	84.162	86.008	85.22	86.13	72.881
1227	100.293	84.984	86.619	85.469	86.089	73.698
1228	99.305	84.413	85.667	84.777	85.64	73.078
1229	100.307	85.169	86.508	85.505	86.549	73.739
1230	99.542	84.357	85.827	85.575	86.589	73.548
1231	100.098	84.588	85.975	85.212	86.7	74.075
1232	100.205	85.117	86.031	85.274	86.451	73.857
1233	100.045	85.196	86.199	85.577	86.459	73.736
1234	99.674	84.883	86.253	85.599	86.467	73.873
1235	100.192	85.011	86.192	85.931	86.522	74.479
1236	99.732	84.702	86.304	85.95	86.143	74.27
1237	99.451	85.132	86.404	85.798	86.207	74.176
1238	99.59	84.923	86.613	85.589	86.497	73.78
1239	100.59	85.153	87.346	86.363	87.173	74.52
1240	100.136	85.413	87.14	86.349	87.018	74.782
1241	100.254	85.54	87.096	86.326	86.638	75.12
1242	100.222	85.998	87.142	86.188	87.307	74.873
1243	100.114	85.255	86.761	85.996	86.703	74.506
1244	100.087	85.262	87.076	86.633	87.06	75.063
1245	99.709	85.35	87.071	86.091	86.907	74.815
1246	100.607	86.083	87.287	86.781	88.004	75.632
1247	100.048	85.81	87.37	86.347	86.512	75.115
1248	99.93	85.983	86.746	86.291	86.788	74.966
1249	100.099	85.609	87.383	86.249	86.982	75.107
1250	99.68	85.614	87.217	86.136	86.923	75.274
1251	99.508	85.463	86.845	85.89	86.865	74.983
1252	99.915	85.531	87.576	86.688	87.492	75.457
1253	100.683	86.582	88.294	87.648	87.514	76.126
1254	100.511	85.829	87.834	87.107	87.449	75.9
1255	100.832	86.178	87.801	87.058	87.024	75.707
1256	100.147	86.458	87.658	86.675	87.048	75.503
1257	100.614	86.526	88.048	86.993	87.585	76.131
1258	99.781	86.272	87.525	86.522	87.118	75.632
1259	99.615	85.906	88.166	86.353	87.061	75.911
1260	100.249	85.718	87.202	86.729	87.662	75.971
1261	100.057	86.615	87.678	86.978	87.609	76.408
1262	100.033	85.987	87.5	87.261	87.246	76.336
1263	100.096	86.376	87.889	87.025	86.859	76.288
1264	100.382	86.02	87.752	86.354	87.314	76.263
1265	99.896	85.853	87.462	86.518	87.448	76.293
1266	100.199	86.287	88.336	87.054	87.223	76.456
1267	99.931	86.166	87.848	86.818	87.567	76.587
1268	100.256	86.381	87.87	86.624	87.766	76.971
1269	99.888	86.161	87.721	87.333	87.544	77.228
1270	100.347	86.136	87.548	86.72	87.331	76.774
1271	100.205	86.106	88.171	86.772	87.763	76.695
1272	100.13	86.161	88.001	86.573	87.8	76.804
1273	100.825	86.729	88.115	87.419	87.983	77.142
1274	100.075	86.17	87.812	87.041	87.949	76.858
1275	100.543	85.825	88.124	86.451	87.525	77.331
1276	99.938	86.238	87.609	86.654	87.581	77.301
1277	99.717	86.247	87.89	86.911	87.44	77.647
1278	99.973	85.609	87.59	87.019	87.943	77.205
1279	100.129	86.552	87.985	87.007	87.452	77.686
1280	100.5	86.374	88.664	87.525	87.974	77.958
1281	100.518	85.925	87.842	87.231	87.85	77.703
1282	99.961	86.702	88.078	87.403	87.54	77.629
1283	100.486	87.011	88.765	87.562	87.7	78.025
1284	100.91	87.418	88.184	87.793	87.829	77.356
1285	100.316	87.38	88.94	87.999	88.255	78.387
1286	99.721	86.669	88.426	87.77	88.009	78.019
1287	100.454	87.78	88.862	87.875	88.045	78.272
1288	99.496	87.078	88.834	87.596	87.855	77.923
1289	100.249	87.668	88.578	87.503	87.996	77.664
1290	100.523	86.779	88.739	88.001	87.445	78.546
1291	99.979	87.091	88.361	87.16	87.57	78.015
1292	99.864	87.392	88.862	86.831	87.445	77.877
1293	100.502	87.516	88.833	88.194	88.05	78.077
1294	99.816	87.391	89.36	87.947	88.27	78.51
1295	100.349	87.706	89.655	88.717	88.086	78.645
1296	100.121	87.819	88.911	88.144	87.743	78.455
1297	99.673	87.37	88.645	88.03	87.479	78.175
1298	100.526	87.431	89.604	88.406	88.538	79.047
1299	99.525	87.133	88.95	87.473	87.336	77.948
		Anowar_SI_NPND_11_RMIT				

Open Researcher and Contributor ID (ORCID) Md. Anowar Hossain: https://orcid.org/0000-0003-2880-6287

Table 12. Supporting information of reflection (%) from 1300 nm to 1400 nm for standardized barium sulphate, *Swietenia Macrophylla* dyed fabric without mordanting, *Swietenia Macrophylla* dyed fabric with mordanting, undyed knitted fabric, raw *Swietenia Macrophylla* and raw *Areca Catechu*.

Wavelength (nm)	Standardized Barium Sulphate	Swietenia Macrophylla dyed fabric without mordanting	Swietenia Macrophylla dyed fabric with mordanting	Undyed knitted fabric	Swietenia Macrophylla	Raw Areca Catechu
1300	100.619	87.734	90.038	88.281	88.136	78.915
1301	100.075	88.189	89.155	88.307	88.381	79.031
1302	100.118	87.788	89.939	88.741	88.312	78.465
1303	100.2	87.811	89.546	88.635	87.816	78.807
1304	100.264	87.708	89.268	88.37	88.226	79.158
1305	100.597	87.632	90.202	89.123	88.77	79.106
1306	99.854	87.51	89.409	88.28	87.964	78.338
1307	99.622	87.534	89.082	88.369	87.96	78.782
1308	99.811	87.721	89.14	88.382	88.051	78.887
1309	99.993	87.406	89.548	88.096	88.221	78.896
1310	100.245	88.38	89.526	88.111	87.796	78.519
1311	99.548	88.082	89.133	87.875	86.957	78.83
1312	100.394	88.518	89.912	88.696	88.446	79.688
1313	99.778	87.663	88.979	88.196	87.687	78.726
1314	99.729	88.252	89.576	88.494	88.083	79.17
1315	99.617	88.127	89.805	88.097	87.593	79.244
1316	100.184	88.022	89.315	88.098	87.978	79.664
1317	99.673	87.388	89.439	88.112	87.74	79.167
1318	100.82	87.699	89.598	88.556	87.655	79.842
1319	99.676	87.4	89.117	88.236	87.622	79.424
1320	100.592	87.554	89.761	88.097	88.172	79.268
1321	100.175	87.744	89.787	88.931	88.105	79.404
1322	99.901	87.863	89.023	88.015	88.501	79.388
1323	100.866	87.895	89.093	88.771	88.027	80.334
1324	100.01	87.185	89.306	88.027	87.557	78.825
1325	100.748	87.922	89.588	89.124	88.442	80.021
1326	100.132	87.441	88.922	88.305	87.561	79.539
1327	100.19	87.532	88.817	88.351	87.797	79.617
1328	99.904	87.785	89.316	88.055	87.711	79.94
1329	101.133	87.954	89.85	88.278	87.726	80.156
1330	100.317	87.956	89.514	87.686	87.069	79.935
1331	99.649	87.101	89.175	87.772	87.416	79.848
1332	100.086	87.168	88.685	87.946	87.173	79.595
1333	100.34	86.962	89.384	87.669	87.138	79.456
1334	100.6	87.639	89.578	87.835	87.305	79.394
1335	99.708	86.727	88.858	87.745	87.333	78.903
1336	99.255	86.454	88.765	87.392	87.103	78.798
1337	100.693	86.955	88.788	87.83	87.365	79.804
1338	100.139	87.097	88.626	87.533	86.875	80.209
1339	100.251	87.184	88.672	87.745	86.932	79.814
1340	100.415	87.041	88.92	87.704	87.241	79.881
1341	100.252	86.739	88.344	87.051	87.19	80.143
1342	99.928	87.156	88.271	87.881	87.096	79.902
1343	99.544	86.257	87.616	86.078	86.38	78.772
1344	99.334	85.433	87.994	86.175	85.324	78.097
1345	100.415	86.526	88.5	87.296	86.612	79.58
1346	99.135	85.613	87.38	86.917	86.438	79.296
1347	100.486	86.096	88.199	86.297	85.922	79.878
1348	99.331	86.057	87.404	86.469	85.646	79.595
1349	100.409	86.45	87.772	86.636	85.869	80.109
1350	100.031	86.074	87.723	86.444	85.465	79.754
1351	99.841	86.183	87.889	86.684	85.775	79.211
1352	100.289	85.621	87.462	86.096	85.886	79.345
1353	99.954	85.678	86.685	85.663	85.617	79.071
1354	100.752	85.486	87.955	86.054	86.251	79.781
1355	101.715	85.428	86.688	86.02	85.328	79.817
1356	99.547	84.837	86.294	84.796	84.531	79.118
1357	99.92	84.603	87.083	85.864	85.142	79.614
1358	101.133	84.312	86.749	84.411	85.227	79.677
1359	100.168	84.378	86.636	85.226	84.995	80.074
1360	101.01	85.207	86.067	84.726	85.24	79.945
1361	100.185	83.796	85.262	84.574	84.999	79.3
1362	99.917	82.74	85.252	82.932	84.259	79.028
1363	99.785	83.867	85.844	83.811	86.002	79.652
1364	100.871	82.032	85.226	83.252	84.08	79.337
1365	99.948	83.123	84.896	83.894	83.903	79.094
1366	99.433	83.012	84.701	83.414	84.092	79.327
1367	100.639	82.743	85.214	83.725	84.818	79.747
1368	100.75	83.249	85.25	83.954	85.227	79.567
1369	99.807	82.881	84.718	83.652	84.573	79.729
1370	100.341	83.795	85.864	84.19	85.249	81.003
1371	100.054	83.474	85.857	83.106	85.546	80.904
1372	100.86	82.951	85.114	83.588	85.888	80.464
1373	99.909	82.811	85.404	84.079	85.543	80.403
1374	100.121	83.335	84.945	84.228	85.578	80.298
1375	99.489	83.6	86.166	84.516	85.684	80.593
1376	100.654	84.952	85.991	85.23	86.134	81.943
1377	99.929	84.151	87.584	84.957	86.65	80.814
1378	100.808	85.018	86.8	85.696	87.086	82.193
1379	100.965	85.98	87.389	86.383	87.623	82.067
1380	99.607	83.229	87.336	85.925	86.562	81.697
1381	99.059	85.119	87.413	84.415	85.731	81.652
1382	100.691	85.249	87.776	86.927	88.829	82.378
1383	100.313	85.371	87.818	86.599	86.379	82.679
1384	99.588	85.273	86.989	85.717	85.963	81.703
1385	99.766	85.284	87.196	85.47	86.941	81.502
1386	101.134	85.176	87.914	86.057	85.975	82.207
1387	99.629	84.07	87.075	85.646	85.334	80.56
1388	100.478	84.39	86.897	85.016	84.932	80.989
1389	101.441	85.109	86.934	85.413	85.474	81.575
1390	100.661	84.849	86.575	86.856	85.951	80.882
1391	100	84.369	86.057	85.142	84.392	79.287
1392	100.472	84.314	87.241	85.763	85.691	80.015
1393	100.853	83.733	86.592	85.376	83.831	79.658
1394	101.597	83.992	85.808	85.526	83.222	80.633
1395	100.221	81.554	85.796	84.665	82.09	77.201
1396	99.796	81.733	84.221	83.261	81.958	77.548
1397	100.767	82.461	84.676	83.557	82.374	77.038
1398	99.53	81.783	84.06	82.488	80.267	76.718
1399	100.821	82.007	84.6	83.293	82.029	77.227
1400	99.977	81.33	83.414	81.694	80.456	76.584
		Anowar_SI_NPND_12_RMIT				

Open Researcher and Contributor ID (ORCID) Md. Anowar Hossain: https://orcid.org/0000-0003-2880-6287

Table 13. CIE L*, a*, b* and RGB values for NPND dyed-coated-printed part of garments placed against multidimensional CBs.

	L*	a*	b*	Red	Green	Blue
Standardized white fabric, a	80	-1	-3	196	203	211
Swietenia Macrophylla dyed fabric, b	51	8	11	153	131	117
Mangifera Indica dyed fabric, c	41	8	17	107	77	50
Terminalia Arjuna dyed fabric, d	58	3	18	155	137	101
Corchorus Capsularis dyed fabric-1, e	65	0	5	148	149	133
Standardized black fabric, f	4	0	0	10	10	12
Camellia Sinensis dyed fabric, g	68	8	21	179	148	117
Corchorus Capsularis dyed fabric-2, h	71	0	-4	185	192	202
Azadirachta Indica dyed fabric, i	65	3	8	153	141	129
Acacia Acuminata dyed fabric, j	65	3	15	172	150	127
Areca Catechu dyed fabric, k	68	8	11	190	164	151
Cinnamomum Tamala dyed fabric, l	77	1	5	188	179	174
Swietenia Macrophylla coated fabric, m	6	9	5	40	21	15
Swietenia Macrophylla printed fabric, n	41	17	23	137	93	68
Swietenia Macrophylla non-printed area of fabric, n	79	0	-1	210	217	225
Corchorus Capsularis coated fabric, o	10	4	9	32	23	14
Corchorus Capsularis printed fabric, p	47	4	17	157	139	103
Corchorus Capsularis non-printed area of fabric, p	71	0	-1	222	221	227
Shorea Robusta Gaertn (stem/bark), CB	36	10	19	117	95	72
Shorea Robusta Gaertn (green leaves), CB	27	-17	25	43	72	44
Shorea Robusta Gaertn (soil bed with dry leaves), CB	37	4	16	108	101	75
Dry wood of Eucalyptus Citriodora, wooden bridge; CB	45	0	4	108	108	101
Dry wood of Bamboo Vulgaris, wooden bridge, CB	32	2	7	130	127	118
Soil background, CB	67	5	28	190	163	116
Bamboo Vulgaris, CB	24	-7	19	46	53	20
Musa Acuminata, CB	38	-5	14	133	136	81

Open Researcher and Contributor ID (ORCID) Md. Anowar Hossain: https://orcid.org/0000-0003-2880-6287
